# Spatiotemporal Organization of PTK7 Diffusion on Cell Surface Facilitates Tumor Invasion and Migration

**DOI:** 10.1002/advs.202517876

**Published:** 2026-03-04

**Authors:** Yaohua Li, Tao Pan, Yu Wang, Sainan Guo, Daiquan Chen, Liu Liu, Liwei Wang, Yang Sun, Weihong Tan

**Affiliations:** ^1^ Institute of Molecular Medicine (IMM) Renji Hospital School of Medicine Shanghai Jiao Tong University Shanghai P. R. China; ^2^ Department of Gastrointestinal Surgery Renji Hospital School of Medicine Shanghai Jiao Tong University Shanghai P. R. China; ^3^ State Key Laboratory of Systems Medicine For Cancer of Oncology Department and Shanghai Cancer Renji Hospital School of Medicine Shanghai Jiao Tong University Shanghai P. R. China; ^4^ Zhejiang Cancer Hospital Hangzhou Institute of Medicine (HIM) Chinese Academy of Sciences Hangzhou P. R. China

**Keywords:** dynamic diffusion, pseudokinases, single‐molecule tracking, tumor invasion and migration

## Abstract

The spatiotemporal dynamics of protein kinase diffusion govern signal activation cascades, thereby modulating fundamental cellular functions. Pseudokinases, catalytically inactive members of the protein kinase superfamily, utilize noncatalytic signaling mechanisms to exert pivotal cellular functions and are frequently dysregulated in human diseases. While nanoscale dynamics of catalytically active receptors regulate signaling integrity, the functional significance of pseudokinase spatial organization remains unknown. Here, using aptamer‐based single‐molecule tracking in living cells, we observed heterogeneous diffusion modes of pseudokinase PTK7 (confined, Brownian, and directed motion). Specifically, spatially PTK7 diffusion coefficients (D) quantitatively correlate with metastatic potential across pancreatic, colorectal and breast cancer cell lines. Functional validation demonstrates that antibody‐mediated PTK7 immobilization suppresses invasion, while Epithelial‐Mesenchymal Transition (EMT) induction accelerates diffusion kinetics to promote metastasis. Crucially, faster PTK7 mobility increases stochastic collision frequency with tyrosine kinase‐like orphan receptor 2 (ROR2), enhancing complex formation to robustly activate the WNT/PCP pathway. Moreover, in patient‐derived primary cells, accelerated PTK7 kinetics positively correlate with invasive and metastatic phenotypes, confirming the clinical relevance of this biophysical regulatory mechanism. This work establishes pseudokinase spatial dynamics as a biophysical regulator of tumor progression, revealing a non‐catalytic paradigm where receptor diffusion kinetics encode cellular behavior through stochastic signaling potentiation.

## Graphical abstract

The spatiotemporal dynamics of protein kinase diffusion govern cellular signaling and functions. Although vital for active receptors, the role of pseudokinase dynamics is unclear. Here, we use single‐molecule tracking to link faster diffusion of pseudokinase PTK7 to increased tumor cell invasion and migration. This establishes PTK7 dynamics as a biomarker for metastatic potential and reveals a new avenue for targeted therapy by controlling kinase dynamics.

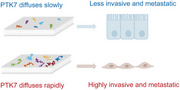

## 1 Introduction

Protein kinases, as integral components of virtually all signaling pathways, control physiological processes and are pharmacological targets [1]. Pseudokinases, catalytically impaired members of the protein kinase superfamily, utilize noncatalytic signaling mechanisms to exert pivotal cellular functions and are frequently dysregulated in human diseases such as cancer [2, 3]. The nanoscale spatial dynamics of catalytically active receptor kinases regulate interactions with ligands and downstream effectors, thereby directly controlling cell signaling outcomes and fundamental cellular functions [4, 5, 6, 7, 8]. However, the cell surface spatiotemporal dynamics of noncatalytic pseudokinases and their functional impacts on cellular processes remain largely unexplored. Consequently, it is essential to resolve pseudokinase dynamics and their mechanistic relationships to cellular functions in native environments with minimal perturbation.

Single‐molecule tracking (SMT) delivers high spatiotemporal resolution and sensitivity for monitoring molecular dynamics in living cells [9]. To preserve native conformation and activity of receptors, developing minimally perturbative labeling probes is essential. Unlike traditional bulky labels such as antibodies or overexpressed protein fusions, aptamers offer a compact alternative with minimal steric interference [10]. Furthermore, aptamers are produced as identical copies via chemical or enzymatic synthesis, enabling more precise dye stoichiometry than antibody labeling—a critical advantage for quantitative nanoscale single‐molecule imaging [11]. Given these advantages, aptamers represent a superior choice for SMT labeling.

Protein tyrosine kinase 7 (PTK7) is a receptor tyrosine kinase (RTK) family member characterized as a pseudokinase lacking catalytic activity [12]. It consistently functions as a co‐receptor, binding to and dimerizing with other catalytically active tyrosine kinase receptors [13]. For instance, PTK7 forms heterodimers with receptors such as orphan receptor 2 (ROR2) within non‐canonical WNT signaling pathways, thereby regulating cell polarity and motility [14, 15, 16]. Abundant evidence has proven that PTK7 is abnormally expressed in solid tumors, such as colorectal [12], non‐small cell lung cancer [17], breast [18], pancreatic tumors [19], and other solid tumors [20, 21, 22], and blood cancers like acute myeloid leukemia [23]. Moreover, high expression levels of PTK7 are closely associated with tumor proliferation [24], invasion and metastasis [14, 22], and therapy resistance [25, 26]. However, PTK7's dynamic diffusion behavior at the cell membrane and its integration of subsequent signals to regulate cellular functions remain largely uncharacterized.

Here, we resolved real‐time PTK7 dynamics on living cell membranes using single‐molecule tracking (SMT) with an Atto647N‐labeled Sgc8c aptamer probe. We revealed heterogeneous PTK7 diffusion characterized by three mobility states (confined, Brownian, directed) and varying coefficients (D). Crucially, PTK7 diffusion coefficients correlated with pancreatic, colorectal, and breast cancer cell invasiveness. Antibody‐mediated inhibition of PTK7 diffusion suppressed highly invasive cell migration, while EMT induction intensified PTK7 dynamics in low‐invasive cells. Enhanced PTK7 mobility promoted ROR2 interactions, robustly activating the WNT/PCP pathway to drive invasion. Specifically, PTK7 molecular motion on primary pancreatic cancer patient‐derived cell surfaces correlates with invasive and metastatic capacity has also been observed. Our findings establish PTK7 spatiotemporal dynamics as regulators of metastatic behavior and provide a mechanistic link between receptor mobility and tumor progression. Monitoring PTK7 diffusion offers translational potential for tumor diagnostic and therapeutic strategies.

## 2 Results and Discussion

### 2.1 Dynamic Tracking of PTK7 on Living Cell Surface

**FIGURE 1:**
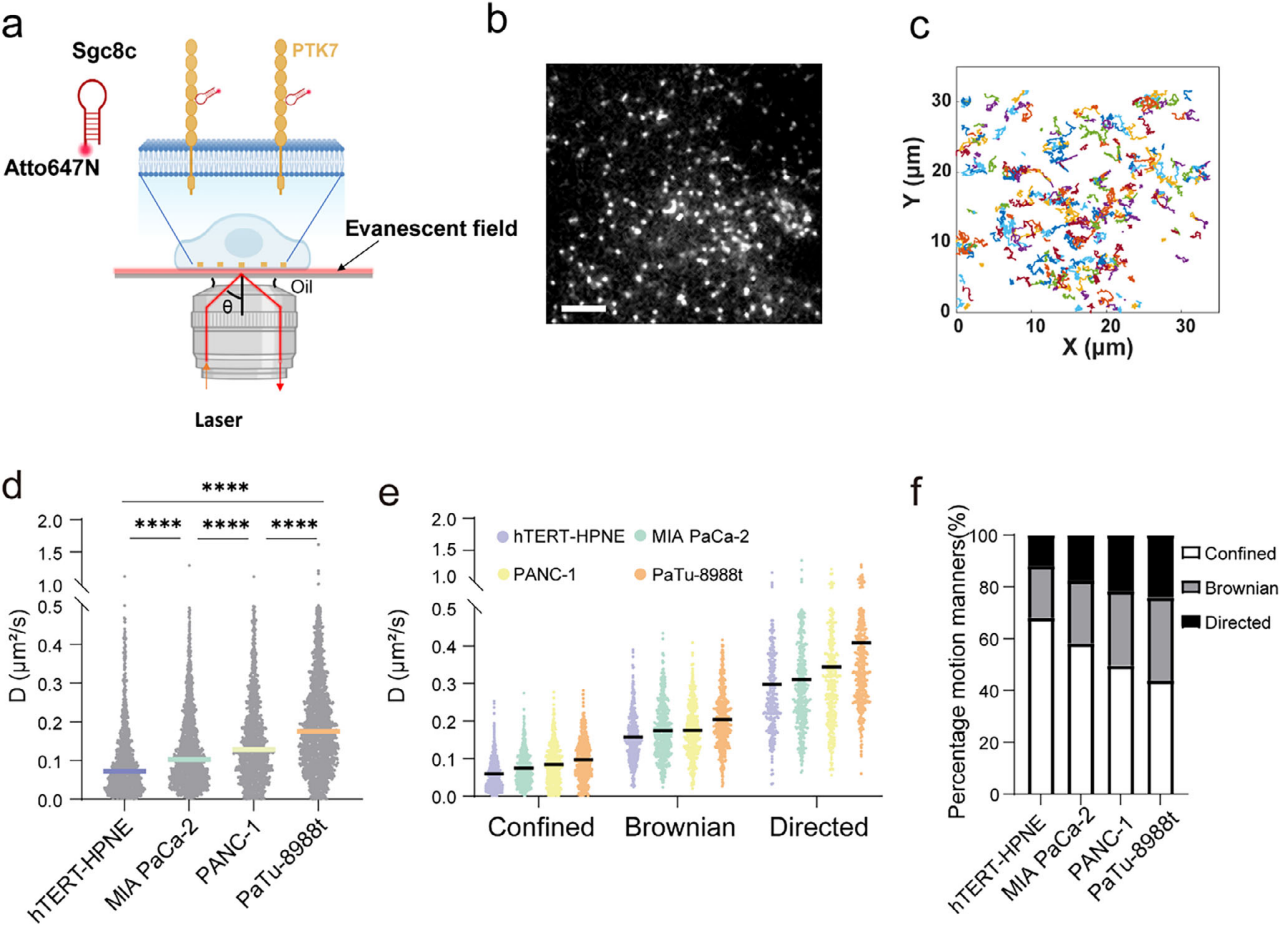
The dynamic diffusion of individual PTK7 molecules on pancreatic cell lines. (a) Schematic diagram illustrating the microscope setup for simultaneous single‐molecule fluorescence imaging of PTK7 on the living cell membrane labeled with Sgc8c aptamer conjugated with fluorescent dye Atto647N. (b) Single‐molecule fluorescence image of PTK7 molecules in PaTu‐8988t cell membrane. (Scale bar: 10 µm). (c) PTK7 trajectories indicated in (b). (d) Diffusion coefficients (D) from individual trajectories analyzed in hTERT‐HPNE, MIA PaCa‐2, PANC‐1 and PaTu‐8988t cells with median marked with colored lines. (e) D values of confined, Brownian, and directed motion among the above four cell lines with median marked in colored lines. (f) Percentage of single trajectories with different types of motion in hTERT‐HPNE, MIA PaCa‐2, PANC‐1, and PaTu‐8988t cells. Significance levels: ns (not significant), *p* > 0.05; *p < 0.05; **p < 0.01; ***p < 0.001; ****p < 0.0001 by one‐way ANOVA with Tukey's multiple comparisons test.

Sgc8c, a well‐characterized 41‐base DNA aptamer that specifically binds PTK7 [27], was conjugated to Atto647N fluorophore with its excellent photostability and fluorescence quantum yield [11] to specifically target and track the dynamic motion of individual PTK7 molecules on living cells. The specific binding of Sgc8c‐Atto647N to PTK7 receptor was confirmed by fluorescence colocalization analysis of PTK7 antibody (Figure S1). Single‐molecule imaging was performed using a total internal reflection fluorescence (TIRF) microscope to specifically visualize PTK7 dynamics on the basal plasma membrane of cells adhering to the coverslips (Figure 1a, Movies S1, S2). Four pancreatic cell lines were studied regarding PTK7 diffusion: a normal pancreatic epithelial cell line (hTERT‐HPNE), two primary pancreatic cancer cell lines (MIA PaCa‐2 and PANC‐1), and PaTu‐8988t, a pancreatic cancer cell line derived from liver metastasis, which can express endogenous PTK7 protein. Pancreatic cell lines were incubated with the Sgc8c‐Atto647N probe for 30 min at 4°C, washed three times with washing buffer, and recovered at 37°C before imaging. The low density of labeled PTK7 was determined to be 0.2 spots/µm^2^ (Figure S2), which is conducive to single‐molecule tracking on the surface of living cells [28]. Single‐molecule fluorescence imaging of the PTK7 receptor on living cells is depicted in Figure 1b. The spot originating from a single fluorophore was validated through a single‐step bleaching process (Figure S3). The dynamic motion of individual PTK7 receptors was continuously monitored at a frequency of 10 Hz with reconstructed trajectories extracted directly from video recordings using a tracking algorithm (Figure 1c) [29].

Mean square displacement (MSD) analysis was performed to assess particle dynamics. MSD quantifies the temporal diffusion rates and classifies the dynamic motion mode based on the diffusion exponent α [30, 31]. The median diffusion coefficients of PTK7 were lowest in normal pancreatic epithelial cells (hTERT‐HPNE: 0.07 µm^2^/s), intermediate in primary pancreatic tumor cells (MIA PaCa‐2: 0.10 µm^2^/s; PANC‐1: 0.13 µm^2^/s), and highest in liver metastasis‐derived pancreatic tumor cells (PaTu‐8988t: 0.18 µm^2^/s), as shown in Figure 1d. The accelerated diffusion of PTK7 in metastatic cells can be attributed to profound alterations in their cellular architecture and membrane environment. As cells acquire metastatic potential, they undergo extensive remodeling of the cytoskeleton [8, 32] and changes in lipid composition [33, 34], both of which are known to govern membrane protein dynamics. Classification of motion modes demonstrated three distinct behaviors: confined, Brownian, and directed motion of PTK7 in living cells. Furthermore, the median D values of confined, Brownian, and directed motion, among these four cell lines, were analyzed from thousands of individual trajectories (Figure 1e). The proportions of confined (68%, 58%, 50%, 44%), Brownian (20%, 24%, 29%, 32%), and directed motion (12%,18%, 21%, 24%) among hTERT‐HPNE, MIA PaCa‐2, PANC‐1, and PaTu‐8988t cells are displayed in Figure 1f. This analysis is robust and does not depend on the specific choice of α thresholds within a reasonable range (Figure S4). It is worth noting that hTERT‐HPNE accounted for the highest proportion in confined motion, while Patu‐8988t had the highest proportion in Brownian and directed motion of PTK7 receptor, indicating that PTK7 molecules in metastasis‐derived PaTu‐8988t cell membrane exhibited more dynamic diffusion and were less restricted. Based on confined trajectories from single‐molecule tracking, we hypothesized that PTK7 movement is restricted by protein clusters [35, 36]. We tested this using dSTORM super‐resolution microscopy, which revealed that PTK7 indeed forms clusters on the cell surface. Critically, these clusters were significantly larger in normal hTERT‐HPNE cells compared to metastatic PaTu‐8988t cells (Figure S5), establishing an inverse correlation between cluster size and protein mobility. Therefore, we conclude that the slower PTK7 dynamics in normal cells are a direct consequence of their containment within larger, more restrictive clusters.

### 2.2 Association Between PTK7 Dynamics and Tumor Cell Invasion and Migration

**FIGURE 2:**
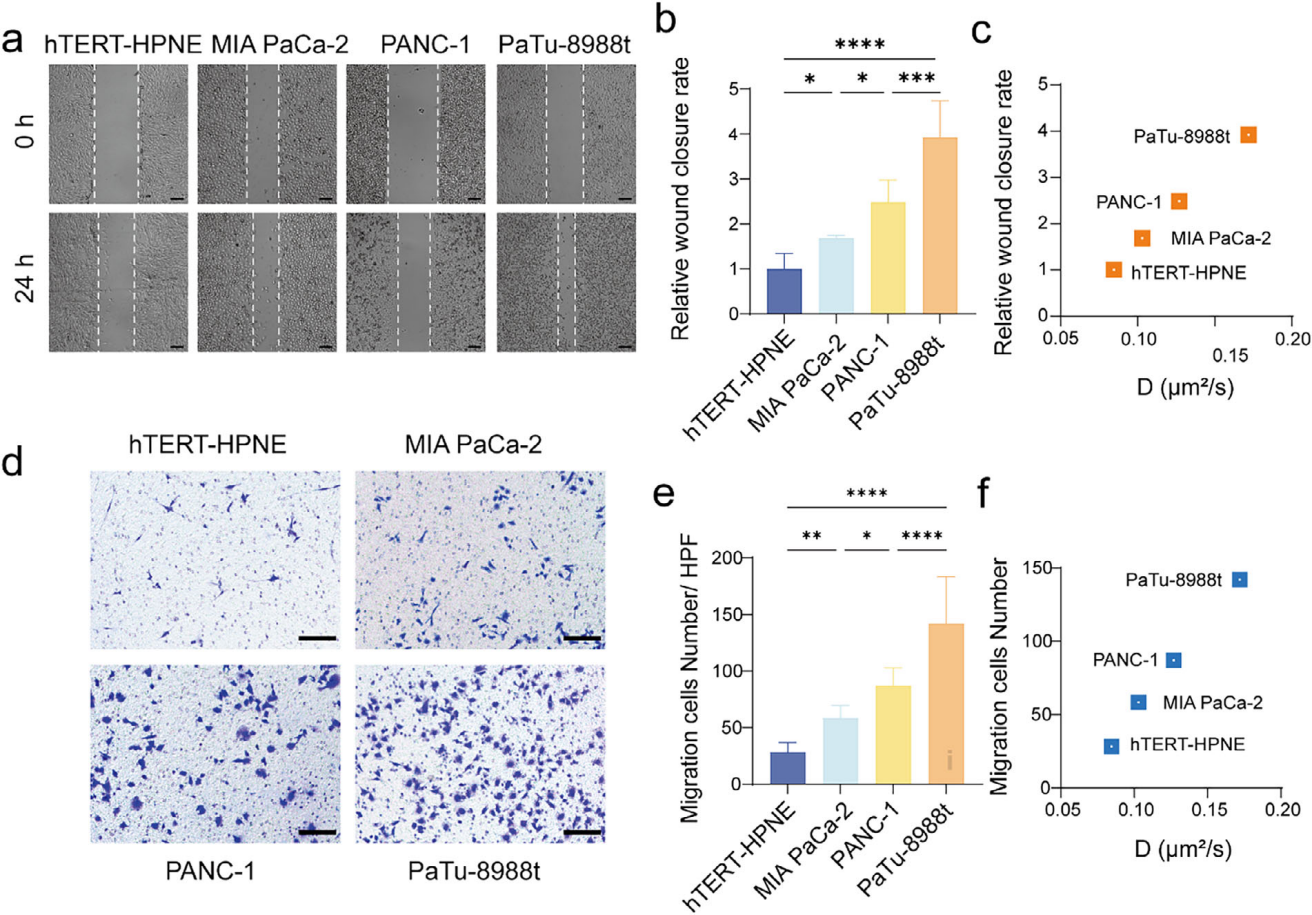
PTK7 dynamic diffusion, cell invasion, and migration. (a) Wound healing assays indicating the mobility of four pancreatic cell lines. Scale bar: 200 µm. (b) Quantitative analysis of the wound healing rate of four pancreatic cell lines for 24 h. (c) Positive dependence of diffusion coefficients (D) and wound closure rate. (d) Images of crystal violet staining demonstrating the invasive capabilities of four pancreatic cancer cell lines. (Scale bar: 100 µm). (e) Quantitative analysis of migration cell numbers of four pancreatic cell lines for 48 h. (f) Positive dependence of diffusion coefficients (D) and migration cell numbers. All data are expressed as mean ± SD (*n* = 3 biological replicates per group from three independent experiments). Significance levels: ns (not significant), *p* > 0.05; *p < 0.05; **p < 0.01; ***p < 0.001; ****p < 0.0001 by one‐way ANOVA with Tukey's multiple comparisons test.

To explore the underlying causes of the differences in PTK7 dynamics on different pancreatic cell surfaces, we examined PTK7 expression levels, cell proliferation, cell cycle, apoptosis, and migration. PTK7 protein level in these four cell lines was analyzed with flow cytometry (Figure S6a) and Western blot (Figure S6c), but no evident correlation between PTK7 protein level and its dynamic motion was observed (Figure S6b,d). To further clarify the influence of PTK7 dynamic motion on cell behavior, cell proliferation, cycle, apoptosis, and migration were studied. Results demonstrated that cell proliferation (Figure S7), cell cycle (Figure S8) and apoptosis (Figure S9) were independent of PTK7 dynamic diffusion. We performed scratch wound healing and transwell assays to demonstrate cellular invasive and migratory capabilities. The wound healing rate of primary MIA PaCa‐2, PANC‐1, and metastatic PaTu‐8988t was 1.7, 2.5, and 4.0 times that of normal epithelial hTERT‐HPNE after incubation for 24 h (Figure 2a,b). The derived percentage of gap areas was positively correlated with the diffusion coefficient of PTK7 receptor in these four living cell lines (Figure 2c). The transwell assay further suggested that the invasion ability of these four cell lines depended on the dynamics of the PTK7 receptor (Figure 2d–f). Similar experimental phenomena were consistently observed in colorectal (Figure S10) and breast (Figure S11) cancer cell lines.

The above experiments fully proved that PTK7 dynamic diffusion in living cells are closely connected to cellular invasion and migration. This correlation was expected because previous reports have shown that the expression and proteolysis of PTK7 were directly linked to tumor cell invasion and migration [37]. Furthermore, PTK7 contributed to the structure and dynamics of cellular protrusions (outward extensions of plasma membrane), thereby facilitating the cell migration process [14].

### 2.3 Functional Validation of PTK7 Diffusion in Cell Migration

**FIGURE 3:**
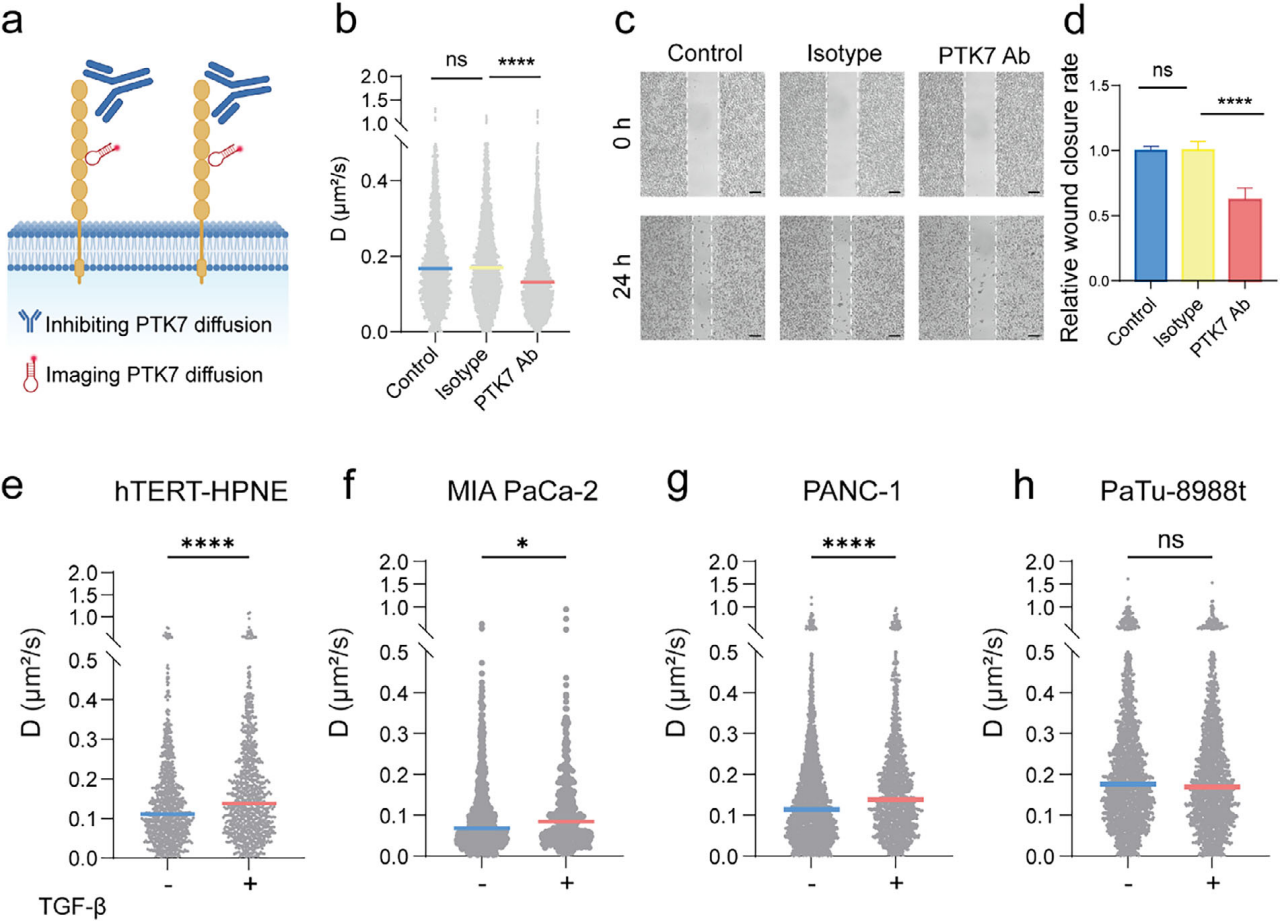
Functional validation. (a) The schematic illustrates the inhibition of PTK7 diffusion through the PTK7 antibody. (b) PTK7 diffusion coefficient (D) was reduced following PTK7 antibody treatment. (c) Wound healing assay demonstrating a significant reduction in the migratory capacity of PaTu‐8988t cells following PTK7 antibody treatment for 24 h. Scale bar: 200 µm. (d) Quantitative analysis of the wound healing rate of PaTu‐8988t cells. (e–h) Distribution of diffusion coefficients (D) and median values (marked with blue and red lines) after TGF‐β treatment in living hTERT‐HPNE, MIA PaCa‐2, PANC‐1 and PaTu‐8988t cells. All data are expressed as mean ± SD (*n* = 3 biological replicates per group from three independent experiments). Significance levels: ns, *p* > 0.05, * *p* < 0.05, ** *p* < 0.01; *** *p* < 0.001, **** *p* < 0.0001 by unpaired two‐tailed Student's *t*‐test.

To further define the functional link between PTK7 dynamics and cellular migration, we perturbed PTK7 dynamics in living cells using antibody blockade strategy [9]. Spatial restriction of PTK7 molecular diffusion (Figure 3a) was achieved through incubation with a commercially sourced anti‐PTK7 monoclonal antibody (1:200 dilution in binding buffer). For single‐molecule tracking, PaTu‐8988t cells were first co‐incubated with an anti‐PTK7 monoclonal antibody and the Sgc8c‐Atto647N aptamer in binding buffer for 30 min at 4°C. Following incubation, unbound antibody and aptamer were removed by washing the cells three times with washing buffer. Then, PTK7 diffusion dynamics were visualized using single‐molecule tracking with TIRFM. Quantitative analysis revealed a significant 22% reduction in the diffusion coefficient of PTK7 following monoclonal antibody treatment compared to untreated and Isotype controls (Figure 3b). Following the antibody‐mediated inhibition of PTK7 molecular diffusion for 24 h, we quantitatively assessed cellular migratory capacity, revealing a closely corresponding 38% reduction in cell migration (Figure 3c,d). We hypothesize that this inhibitory effect could arise from two non‐mutually exclusive mechanisms: (1) a steric hindrance effect, where the large size of the antibody physically impedes the movement of PTK7 within the crowded membrane environment; (2) a functional blocking effect, where the antibody binding to the extracellular domain of PTK7 prevents its interaction with essential ligands or co‐receptors required for downstream signaling and cell movement. This antibody‐mediated constraint of PTK7 mobility directly establishes a functional link between receptor diffusion dynamics and cellular migration, demonstrating that impaired molecular motion reduces metastatic cell motility.

Cancer cell invasion and migration in vivo are strongly linked to Epithelial‐Mesenchymal Transition (EMT), a process whereby epithelial cells gain mesenchymal characteristics to promote metastasis and invasiveness [38, 39]. Using TGF‐β to induce EMT in pancreatic cancer cells [40, 41], we tested whether PTK7 motion dynamics are linked to enhanced migration. Following 48‐h TGF‐β (10 ng/mL) treatment, immunofluorescence confirmed EMT progression, evidenced by decreased E‐cadherin and increased vimentin expression in MIA PaCa‐2, PANC‐1, and PaTu‐8988t cells (Figure S12). TGF‐β treatment significantly enhanced the migratory capacity of MIA PaCa‐2 and PANC‐1 cells (Figure S13a,b), but had minimal effect on PaTu‐8988t cells (Figure S13c), consistent with their intrinsically high baseline metastatic potential and EMT marker expression (Figure S12). We analyzed the dynamics of PTK7 at 24, 48, and 72 h post‐treatment showed in Figure S14. The results show a time‐dependent effect of TGF‐β on PTK7 dynamics. Specifically, the diffusion coefficients (D) of PTK7 progressively increased compared to the untreated control group, with activation kinetics peaking around 48 h. PTK7 D values increased in hTERT‐HPNE, MIA PaCa‐2, and PANC‐1 cells after 48‐hour TGF‐β‐induced EMT and enhanced migration (Figure 3e–g). In contrast, PaTu‐8988t PTK7 D values remained largely unchanged (Figure 3h). Collectively, these bidirectional perturbations demonstrate that PTK7 lateral diffusion dynamics are mechanistically coupled to cellular migration.

### 2.4 Mechanism of PTK7 Dynamics in WNT Signaling Pathway Activation

**FIGURE 4:**
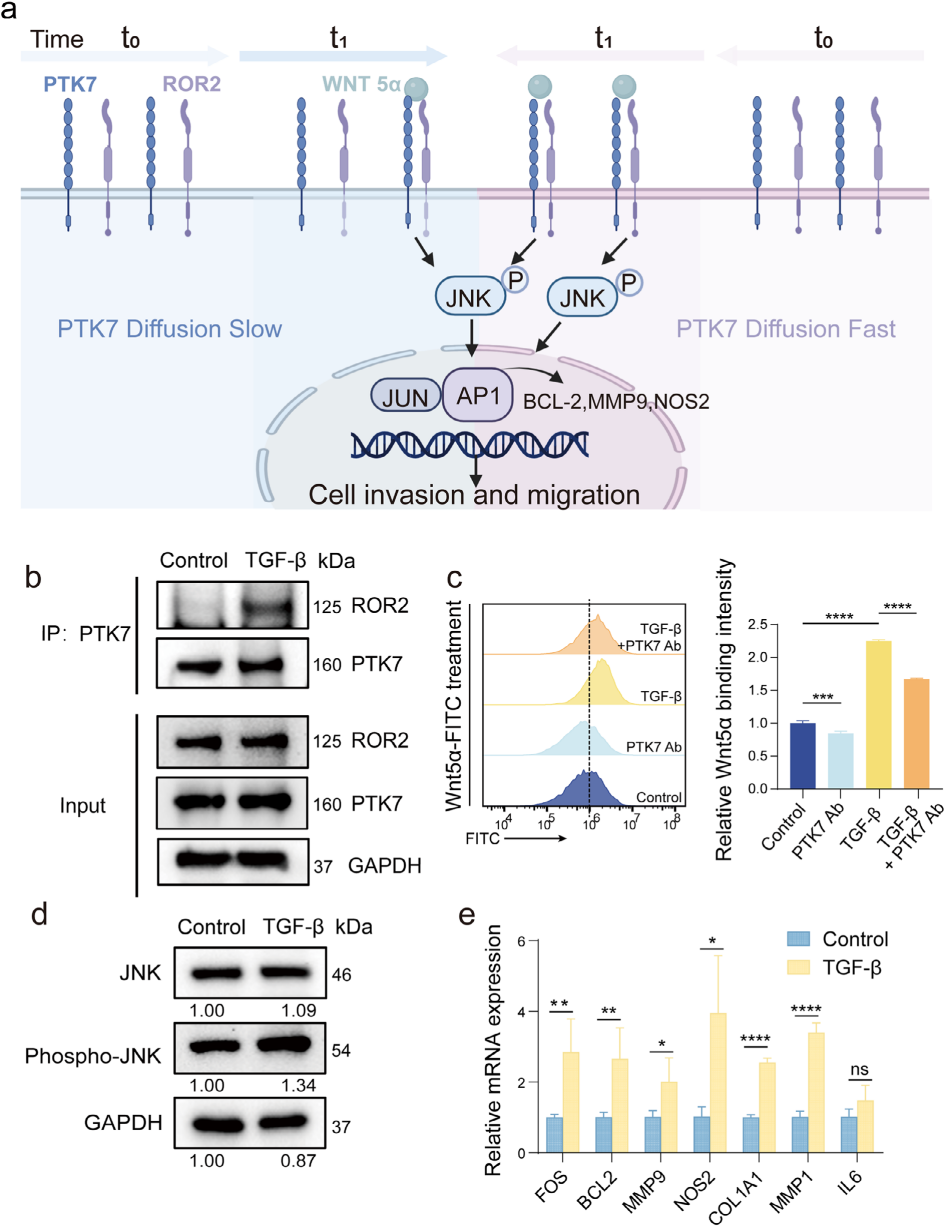
The impact of PTK7 dynamic motion on signaling pathways. (a) Schematic showing correlation between the dynamic motion of PTK7 receptors and cell mobility. (b) Co‐IP assay showing the increasing interactions between PTK7 and ROR2 treated with TGF‐β. (c) Quantification of Wnt5α binding to PANC‐1 cells by flow cytometry. Cells were treated as indicated: Control, PTK7 antibody (PTK7 Ab), TGF‐β, and a combination of TGF‐β + PTK7 Ab. (d) Phosphorylation level of JNK protein after TGF‐β induction. (e) Level of transcription factors in the WNT/PCP signaling pathway. All data are expressed as mean ± SD (*n* = 3 biological replicates per group from three independent experiments). Significance levels: ns, *p* > 0.05, * *p* < 0.05, ** *p* < 0.01; *** *p* < 0.001, **** *p* < 0.0001 by unpaired two‐tailed Student's *t*‐test.

PTK7 is implicated in WNT signaling, particularly the non‐canonical pathway associated with cell migration. Specifically, stimulation by the non‐canonical ligand Wnt5α enables physical and functional interaction between the PTK7 extracellular domain and ROR2, leading to activation of c‐Jun N‐terminal kinase (JNK) signaling and initiation of cellular motility [3]. We propose a mechanism whereby enhanced diffusion dynamics of PTK7 increase the stochastic collision probability with ROR2, amplifying JNK pathway activation and ultimately driving increased cell invasion and migration (Figure 4a). First of all, to determine if the pro‐migratory effect of accelerated PTK7 dynamics is dependent on ROR2, we specifically knocked down ROR2 using siRNA in PaTu‐8988t cells exhibiting high PTK7 mobility. The depletion of ROR2 significantly suppressed the enhanced cell migration, confirming that ROR2 is a crucial mediator of PTK7's function in promoting cell motility (Figure S15).

To further investigate the interaction between PTK7 and ROR2, we first performed molecular docking, which predicted a potential interaction between these proteins (Figure S16). To validate this prediction, we conducted co‐immunoprecipitation (Co‐IP) assays in PANC‐1 cell lysates using an anti‐PTK7 antibody. This interaction was also observed in MIA PaCa‐2 and PaTu‐8988t cells (Figure S17). These assays confirmed that PTK7 and ROR2 form a complex. Significantly, TGF‐β treatment, which enhances PTK7 diffusion dynamics, increased the amount of this PTK7‐ROR2 complex in PANC‐1 cells (Figure 4b). Importantly, TGF‐β treatment did not alter the cellular protein levels of PTK7 (Figure S18) or ROR2 (Figure S19), indicating that the increased complex formation is attributable to enhanced PTK7 diffusion dynamics rather than changes in protein expression. Consistent with the increased PTK7‐ROR2 complex formation, the binding of Wnt5α to PANC‐1 cells (Figure 4c), as well as to MIA PaCa‐2 and PaTu‐8988t cells (Figure S20a,b), increased following TGF‐β induction. To directly address this causality between PTK7 diffusion and Wnt5α binding, we investigated whether antibody‐mediated immobilization of PTK7 would block the TGF‐β‐induced increase in Wnt5α binding. The result suggested that immobilizing PTK7 with the antibody significantly reduced Wnt5α binding, even in the presence of TGF‐β stimulation (Figure 4c). This finding provides strong evidence that the increased mobility of PTK7 is a prerequisite for enhanced Wnt5α binding. Immobilizing PTK7 prevents this crucial first step, thus abolishing the downstream increase in ligand binding. Besides, we have also investigated the impact of direct Wnt5α activation on PTK7 dynamics and cell function. While direct Wnt5α treatment failed to alter the diffusion coefficient of PTK7, it significantly promoted cell invasion and migration (Figure S21). This contrasts with the effect of TGF‐β, suggesting that the observed changes in PTK7 dynamics are a specific hallmark of the EMT process rather than a direct consequence of ligand binding.

Further supporting WNT/PCP pathway activation, TGF‐β induction increased the phosphorylation of key pathway components, including JNK (Figure 4d), ROR2, and Dishevelled2 (Dvl2) (Figure S22a,b). Finally, TGF‐β treatment upregulated the expression of WNT/PCP target genes (FOS, BCL2, MMP9, NOS2, COL1A1, MMP1, and IL6), as measured by real‐time PCR (Figure 4e). Crucially, consistent with the activation of this pro‐migratory pathway, TGF‐β induction significantly enhanced the invasion and migration capabilities of PANC‐1 and MIA PaCa‐2 cells (Figure S13). This result suggests that the increased PTK7 diffusion promotes the formation of functional PTK7‐ROR2 heterodimers, leading to greater activation of the WNT/PCP signaling pathway.

Taken together, these results demonstrate that enhanced PTK7 diffusion dynamics, promoted by TGF‐β induction, increase its interaction and complex formation with ROR2. This complex, in turn, enhances Wnt5α binding, activates the WNT/PCP signaling pathway (evidenced by increased phosphorylation of JNK, ROR2, and Dvl2), and elevates the expression of downstream target genes, ultimately promoting cell invasion and migration.

### 2.5 Clinical Potential of PTK7 Kinetics in Monitoring Cancer Metastasis

To comprehensively characterize the spatiotemporal dynamics of PTK7 diffusion under pathophysiologically relevant conditions, we established a real‐time monitoring platform using primary cells derived from pancreatic cancer patients. Three patient‐derived primary pancreatic cancer cells (PAAD‐23020, PAAD‐22001, and PAAD‐21010) were isolated from surgical tumor tissues, with PTK7 expression confirmed by IHC (Figure S23).

**FIGURE 5:**
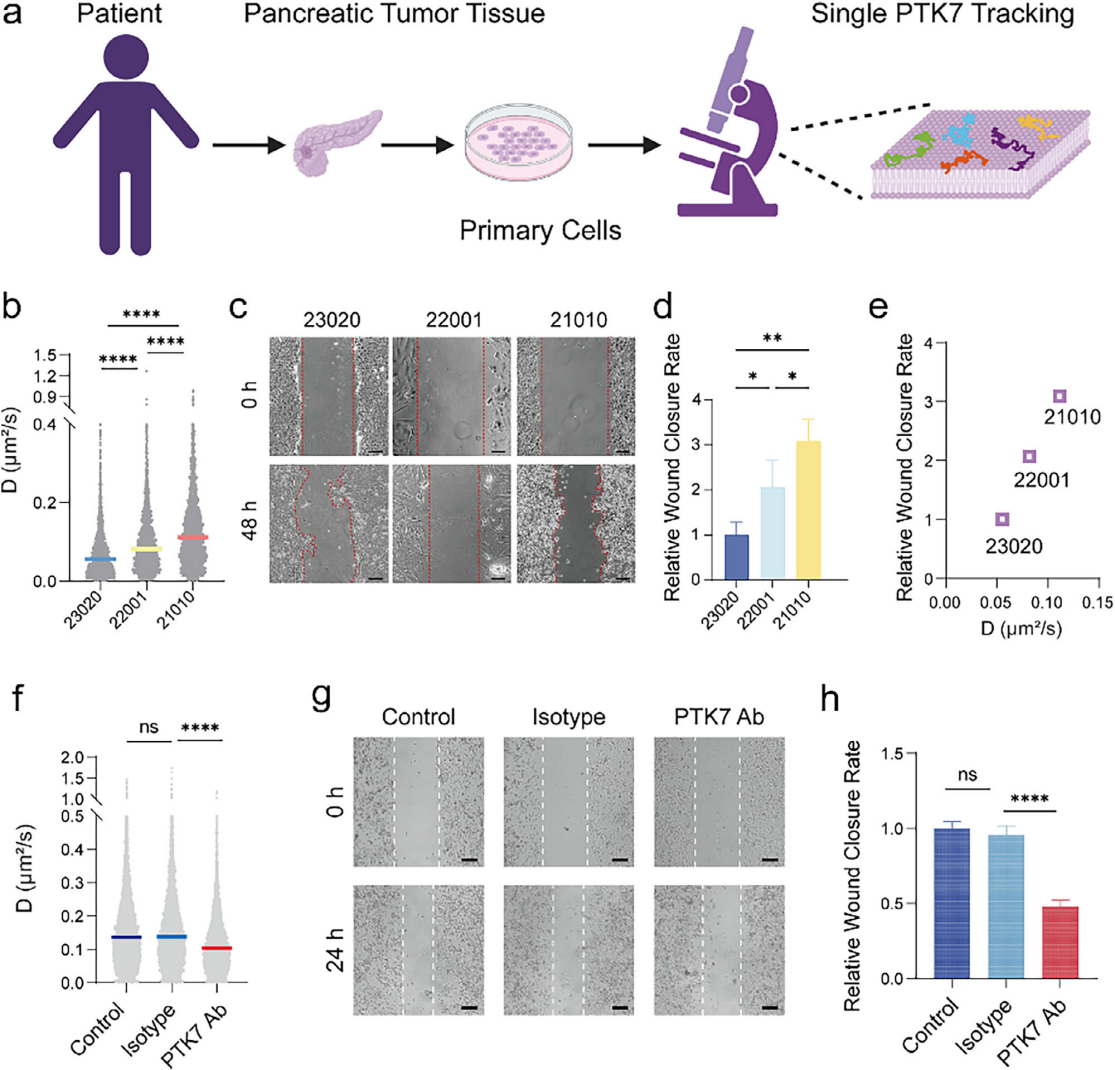
PTK7 dynamics and cell invasion and migration across primary pancreatic cancer cells. (a) The schematic illustrates tracking of the PTK7 receptor on pancreatic cancer patients' cell surfaces. (b) Diffusion coefficients (D) of PTK7 molecules in primary pancreatic cancer cells. The analysis for each cell line is based on a minimum of 1000 diffusion trajectories. (c,d) The mobility of primary pancreatic cancer cells was indicated by wound healing assays and quantitative analysis of the wound healing rate of primary pancreatic cancer cells for 48 h. (e) Positive dependence of diffusion coefficients (D) and wound closure rate. (f) Treatment of primary PAAD‐21010 cells with a PTK7‐specific antibody resulted in a significant reduction in the diffusion coefficient (D) of PTK7. (g,h) The significant inhibition of cell migration in a scratch wound healing assay by antibody‐induced immobilization of PTK7. Scale bar: 200 µm. All data are expressed as mean ± SD (*n* = 3 biological replicates per group from three independent experiments). Significance levels: ns (not significant), *p* > 0.05; *p < 0.05; **p < 0.01; ***p < 0.001; ****p < 0.0001 by one‐way ANOVA with Tukey's multiple comparisons test.

Leveraging single‐molecule tracking (SMT) technology, live primary cells cultured in confocal dishes were labeled with Sgc8c‐Atto647N probes under standardized conditions (Figure 5a). Quantitative analysis revealed that PTK7 exhibited differential diffusion kinetics across patients: PAAD‐21010 cells showed the highest diffusion coefficient (0.11 µm^2^/s) (Figure 5b), which correlated with significantly enhanced migration capacity compared to PAAD‐23020 and PAAD‐22001 cells (Figure 5c,d). Thus, PTK7 dynamics positively correlate with motile behavior in primary cell systems (Figure 5e). Critically, this real‐time dynamic profiling demonstrated that PTK7 diffusion kinetics serve as a functional indicator of cellular invasiveness.

To further investigate the therapeutic potential of targeting PTK7 dynamics, we extended our analysis to clinically relevant primary pancreatic cancer cells derived from patient PAAD‐21010, which exhibit high invasive and migratory capabilities. Treatment with a PTK7 antibody, which restricts the lateral diffusion of PTK7 on the plasma membrane, resulted in a significant reduction in cell motility. Single‐molecule tracking confirmed that the antibody treatment decreased the PTK7 diffusion coefficient by approximately 25% (Figure 5f). Consistently, functional assays showed a corresponding ∼50% reduction in the cellular migration rate (Figure 5g,h). This provides direct evidence linking PTK7's molecular dynamics to the migratory potential of patient‐derived cancer cells.

However, we acknowledge that the findings from our patient‐derived cells (*n* = 3) are preliminary. While they serve as a critical proof‐of‐concept, future studies with a larger and more diverse patient cohort are essential. By expanding our cohort and incorporating long‐term follow‐up data, we will be positioned to not only validate these initial results but also to perform robust correlational analyses between PTK7 dynamics and key clinical parameters, including time to recurrence or metastasis, treatment response, and overall survival. This will be crucial for establishing the broader clinical applicability of PTK7 dynamics as a potential biomarker.

## 3 Conclusion

In summary, the dynamic motion of PTK7 in the living cell membrane is associated with cellular invasion and migration by increasing interaction with co‐receptor ROR2 to activate cell mobility‐related WNT/PCP signaling pathways. A single PTK7 receptor was labeled with the Sgc8c‐Atto647N probe, enabling the real‐time and in situ imaging of PTK7 diffusion through single‐molecule tracking. Correlation between increasing diffusion coefficients of PTK7 and higher cellular migration was investigated in pancreatic, colorectal, and breast tumor cell lines. Inhibition of the dynamic diffusion of PTK7 molecules significantly suppresses the migration of highly invasive pancreatic cancer cells. Furthermore, the diffusion coefficient of PTK7 molecules markedly rose along with the augmentation of cellular invasive and migratory capabilities. This phenomenon was also observed in primary cells from patients with pancreatic cancer, as well as in breast and colorectal cancer cell lines. Moreover, the present study provides a new biophysical indicator to assess the invasive and metastatic capabilities of tumor cells with potential clinical application. It also reveals a promising potential tumor‐targeted therapy by precisely regulating the dynamic behavior of RTK molecules in living cells.

## Author Contributions

Y.H.L. and Y.W. conceived and designed the research. Y.H.L. performed single‐molecule tracking experiments, analyzed the data, and wrote the manuscript. T.P. performed cellular experiments. S.N.G. was responsible for protein quantification analyses. D.Q.C. and L.L. handled partial data analysis. W.H.T., Y.S., and L.W.W. provided supervision.

## Conflicts of Interest

The authors declare no conflicts of interest.

## Supporting information




**Supporting File 1**: advs74566‐sup‐0001‐SuppMat.docx.

**Supporting File 2**: advs74566‐sup‐0002‐MovieS1.avi.

**Supporting File 3**: advs74566‐sup‐0003‐MovieS2.avi.

## Data Availability

The data that support the findings of this study are available from the corresponding author upon reasonable request.
