# Supplementary material for: Spatiotemporal Organization of PTK7 Diffusion on Cell Surface Facilitates Tumor Invasion and Migration
Source: Adv Sci (Weinh). 2026 Mar 4;13(26):e17876. doi: 10.1002/advs.202517876 (PMC13159131; doi:10.1002/advs.202517876)
Supplement: Supplementary file 1 — Supporting File 1: advs74566‐sup‐0001‐SuppMat.docx. [file ADVS-13-e17876-s001.docx]

Supporting Information

**Spatiotemporal Organization of PTK7 Diffusion on Cell Surface Facilitates Tumor Invasion and Migration**

Yaohua Li^[1]#^, Tao Pan^[2]#^, Yu Wang^[3]#^, Sainan Guo^[1]^, Daiquan Chen^[1]^, Liu Liu^[1]^, Liwei Wang^[3]^, Yang Sun^[1]^*, Weihong Tan^[1], [4]^*

[1] Institute of Molecular Medicine (IMM), Renji Hospital, School of Medicine, Shanghai Jiao Tong University, P. R. Shanghai, China.

[2] Department of Gastrointestinal Surgery, Renji Hospital, School of Medicine, Shanghai Jiao Tong University, Shanghai, P. R. China.

[3] State Key Laboratory of Systems Medicine for Cancer of Oncology Department and Shanghai Cancer Institute, Renji Hospital, School of Medicine, Shanghai Jiao Tong University, Shanghai, P. R. China.

[4] Zhejiang Cancer Hospital, Hangzhou Institute of Medicine (HIM), Chinese Academy of Sciences, Hangzhou, P. R. China.

Correspondence: Weihong Tan, email: tan@hnu.edu.cn

Yang Sun, email: suny1989@sjtu.edu.cn

**Table of Contents**

**1. Materials and Methods**

1.1 Chemicals

1.2 Cell Lines and Cell Culture

1.3 Preparation Cells Labeling with Sgc8c-Atto674N Probe

1.4 Microscope Apparatus

1.5 Single PTK7 Molecule Imaging in Living Cells

1.6 Single-Molecule Tracking Analysis

1.7 Statistical Analysis

1.8 Super-resolution Imaging and Analysis

1.9 The Expression of PTK7 Determined by Flow Cytometry

1.10 Cell Proliferation Assay

1.11 Cell Cycle Assay

1.12 Cell Apoptosis Assay

1.13 Wound Healing Assay

1.14 Transwell Migration Assay

1.15 TGF-β Treatment for EMT Process

1.16 EMT Marker Detection

1.17 PTK7 Antibody Inhibition Assay

1.18 Western Blot Analysis

1.19 The Molecular Docking Between PTK7 and ROR2

1.20 Immunoprecipitation Assay

1.21 siRNA-Mediated ROR2 Silencing

1.22 Wnt5α Binding with PTK7 and ROR2

1.23 qRT-PCR

1.24 Immunohistochemistry Analysis

**2. Supplementary Figures**

Figure S1. Specific Binding Between Sgc8c-Atto674N and PTK7

Figure S2. Quantitative Analysis of PTK7 Cell Surface Label Density

Figure S3. Single-Molecule Photobleaching

Figure S4. Classification of PTK7 Diffusion Dynamics under Varying Anomalous Diffusion Exponent (α) Thresholds

Figure S5. STORM Imaging of PTK7 on Basal Membrane

Figure S6. PTK7 Level in Pancreatic Cell Lines

Figure S7. Cell Proliferation in Pancreatic Cell Lines

Figure S8. Cell Cycle in Pancreatic Cell Lines

Figure S9. Cell Apoptosis in Pancreatic Cell Lines

Figure S10. PTK7 Dynamics and Colorectal Cell Migration

Figure S11. PTK7 Dynamics and Mammary Cell Migration

Figure S12. Detection of EMT Marker Expression

Figure S13. Cell Migration Treated with TGF-β

Figure S14. TGF-β Induces Time-dependent Change in PTK7 Diffusion

Figure S15. Effect of ROR2 knockdown on PANC-1 cell motility

Figure S16. PTK7 and ROR2 Docking Prediction

Figure S17. CO-IP Assay of PTK7 and ROR2

Figure S18. PTK7 Protein Level After TGF-β Treatment

Figure S19. ROR2 Protein Level After TGF-β Treatment

Figure S20. Binding Ability of Wnt5α after TGF-β Treatment

Figure S21. Effects of Wnt5α on PTK7 Diffusion and Cell Migration

Figure S22. Protein Phosphorylation in WNT/PCP Pathway

Figure S23. PTK7 Levels in Patients’ Tissues

**3. Supplementary Movies**

*Movie S1. SMI of PTK7 in PaTu-8988t*

*Movie S2. PTK7 Particle Tracking*

**4. References**

**Materials and Methods**

**Chemicals**

Atctaactgctgcgccgccgggaaaatactgtacggttaga-Atto647N was purchased from Sangon Biological Engineering Technology Co. Ltd. (Shanghai, China). Vimentin antibody, PTK7 immunoprecipitation antibody, and ROR2 antibody were obtained from Cell Signaling Technology (CST). Alexa Fluor 488-conjuged PTK7 antibody was purchased from Novus Biologicals, LLC. Alexa Fluor 647 goat anti-mouse IgG was obtained from Beyotime Biotechnology (Shanghai, China). TGF-β1 protein was purchased from MedChemExpress, LLC.

**Cell Lines and Cell Culture**

The following authenticated human cell lines were used in this study, with Research Resource Identifiers (RRIDs) and suppliers indicated: hTERT-HPNE (RRID: CVCL_C466; supplier: Wuhan Pricella Biotechnology Co., Ltd.), PANC-1 (RRID: CVCL_0480; supplier: Wuhan Pricella Biotechnology Co., Ltd.), PaTu-8988t (RRID: CVCL_1847; supplier: Wuhan Pricella Biotechnology Co., Ltd.), NCM460 (RRID: CVCL_0460; supplier: Wuhan Pricella Biotechnology Co., Ltd.), HCT116 (RRID: CVCL_0291; supplier: Wuhan Pricella Biotechnology Co., Ltd.), SW620 (RRID: CVCL_0547; supplier: Wuhan Pricella Biotechnology Co., Ltd.), MCF-7 (RRID: CVCL_0031; supplier: Wuhan Pricella Biotechnology Co., Ltd.), MDA-MB-231 (RRID: CVCL_0062; supplier: Wuhan Pricella Biotechnology Co., Ltd.), MIA PaCa-2 (RRID: CVCL_0428; supplier: Meisen Chinese Tissue Culture Collection Co., Ltd.), and MCF10A (RRID: CVCL_0598; supplier: Meisen Chinese Tissue Culture Collection Co., Ltd.). Primary pancreatic cancer cells (PAAD-23020, PAAD-22001, and PAAD-21010) were obtained from Precedo Pharmaceuticals Co., Ltd. under Institutional Ethics Committee approval (Cancer Hospital, Chinese Academy of Sciences, Hefei; Approval No. PJ-KY2022-005) and with written informed consent from all participants or next of kin. All cell lines and primary cultures were STR-authenticated and tested mycoplasma-negative throughout the study.

hTERT-HPNE, MIA PaCa-2, PANC-1, PaTu-8988t, NCM460, HCT116, SW620, MCF-7, and MDA-MB-231 cell lines were cultured in Dulbecco's Modified Eagle Medium (DMEM, GIBCO), containing 10% FBS and 1% penicillin-streptomycin solution (100 µg/mL). MCF10A cells were cultured in Dulbecco's Modified Eagle Medium/F12 (DMEM/F12, HyClone) with 5% Horse Serum (HyClone), 20 ng/mL EGF (Sigma), 0.5 μg/mL Hydrocortisone (Sigma), 100 ng/ml Cholera toxin (Sigma), 10 μg/mL Insulin (Sigma), 100 μg/mL Penicillin (Sigma) and 100 μg/mL Streptomycin (Sigma). Primary pancreatic cancer cells were cultured in special medium supported by Precedo Pharmaceuticals Co. Ltd. All cells were cultured at 37°C under an atmosphere of 5% CO_2_.

**Labeling Cells with Sgc8c-Atto674N Probe**

1×10^5^ cells were plated in glass bottom 35 mm confocal dishes and then cultured at 37°C with 5% CO_2_ atmosphere. When the cells reached 80% confluence, binding buffer was added to the confocal dishes (DPBS with 4.5 g/L glucose, 5 mM of MgCl_2_, 0.1 mg/mL of yeast tRNA and 1 mg/mL BSA), along with 0.02 nM Sgc8c-Atto674N probe. Dishes were kept at 4°C for 40 minutes to allow sufficient binding of the probe with PTK7 in living cells. Then cells were washed with washing buffer (DPBS supplemented with 4.5 g/L glucose and 5 mM MgCl_2_) three times to remove unbound probe.

**Microscope Apparatus**

Single-molecule tracking of PTK7 receptors in living cells was performed with an Olympus IX83 inverted fluorescence microscope with a 100x oil immersion objective (N.A. =1.49) in a dark room. The Olympus IX83-ZDC module can stabilize vertical focus. The emitted photons were collected by a back-illuminated scientific complementary metal-oxide-semiconductor (sCMOS) camera (Prime 95B, 1024 × 1024 pixels, 11 μm per pixel).

Hoechst 33342 and Alexa Fluor 647-PTK7 antibody were excited by a 405 and 640 nm laser (Toptica iChrome MLE, Germany), respectively. Colocalization analysis primarily used ImageJ (Fiji) with the Coloc 2 plugin. Immunofluorescence images of E-cadherin and vimentin were also imaged using the Nikon Ti2-E microscope, and Hoechst 33342, Alexa Fluor 488-E-cadherin antibody and Alexa Fluor 594-vimentin antibody were excited by a 405, 488, and 561 nm laser, respectively. The fluorescence images were recorded by the sCMOS camera (Photometrics Prime 95B, 1200 × 1200 pixels, 11 μm per pixel, USA).

**Single PTK7 Molecule Imaging in Living Cells**

Living cells labeled with Sgc8c-Atto674N were imaged in DMEM complete growth medium and placed in a live cell microscope incubator at 37°C and 5% CO_2_. Irradiation via Total Internal Reflection (TIR) was utilized. In all cell lines, PTK7 dynamic diffusion was recorded with 30 ms exposure time and attenuated excitation: 60% of maximum power for 647 nm lasers to acquire high signal-to-noise ratio single-molecule fluorescence images. Movies at 300 fps of each dish were acquired within a 100 ms period to minimize photobleaching as much as possible. Each dish with living cells was recorded for three regions of interest (ROIs), and the same cultured cells were imaged for analysis. Prior to each acquisition, the sample was maintained in darkness for 3 minutes to ensure consistent initial conditions.

**Single-Molecule Tracking Analysis**

The trajectories of single PTK7 molecules were obtained with ImageJ using the Trackmate plugin [29, 42]. In brief, the algorithm identifies the locations of intensity peaks, which represent individual fluorescent spots, in each frame based on user-set parameters for size and brightness. These locations are then connected to form trajectories with a maximum allowable movement between frames as specified by the user to match the sample's characteristics. Moreover, the algorithm permits a gap of up to one frame in trajectory reconstruction to account for the possibility that a tracked molecule might briefly escape detection for a single frame.

Mean Square Displacement (MSD) analysis was performed with in-house MATLAB codes to analyze the dynamic heterogeneity and anomalous diffusion of PTK7 receptors. The MSD of each trajectory was calculated according to the following equation:


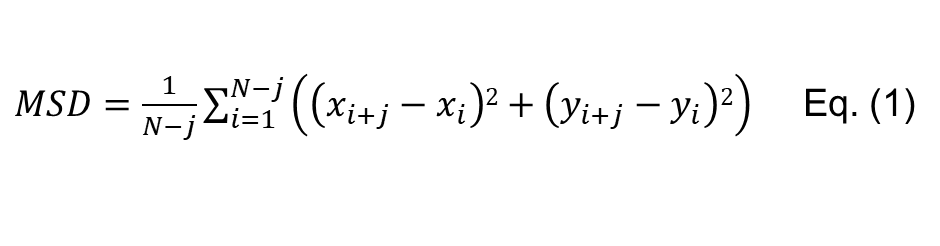


Only trajectories longer than 15 frames were further analyzed and considered the initial one-third portion of the trajectory. The diffusion coefficients (D_t_) for short time intervals were derived from a linear fit applied to the initial five data points of the MSD curves by the following equation^29^:


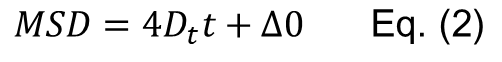


where △_0_ denotes the MSD offset at zero time lag. The distribution of D_t_ generally stemmed from analyzing thousands of individual trajectories across various cells, and the median of diffusion coefficients (D_t_) represents diffusion characteristics of particular cell lines.

The MSD curves depicted in Figure 1d were fitted based on a general anomalous diffusion model characterized by a power law relationship [43, 44].


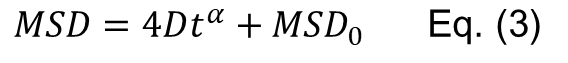


where α is the anomalous diffusion exponent, and MSD_0_ represents a constant offset. The exponent α is determined with the nonlinear fitting of the first fifteen points by Equation 3, which also corresponds to the slope of the MSD curve in the log–log plot. The α values were employed to categorize the types of diffusion (0.9<α<1.1, Brownian; 0<α<0.9, confined; α>1.1, directed motion).

**Statistical Analysis**

All quantitative data were expressed as mean ± standard deviation (SD) from at least three independent experiments. Before statistical analysis, data were assessed for normal distribution using the Shapiro-Wilk test and for homogeneity of variances using Levene's test. For comparisons involving only two groups, a two-tailed unpaired Student’s t-test was applied. For comparisons involving more than two groups, a one-way analysis of variance (ANOVA) was performed. If the ANOVA result was significant, it was followed by Tukey's multiple comparisons test to identify specific group differences. A P-value less than 0.05 was considered to indicate statistical significance. Statistical analyses were conducted using GraphPad Prism version 9.0 (GraphPad Software, La Jolla, CA). In figures, significance is indicated as follows: *P < 0.05, **P < 0.01, ***P < 0.001, and ****P < 0.0001.

**Super-resolution Imaging and Analysis**

The human pancreatic ductal epithelial cell line hTERT-HPNE and the pancreatic cancer cell line PaTu-8988t were used for STORM imaging. Cells were seeded onto small coverslip (22 mm x 22 mm) in 6-well plate and cultured until they reached 60-70% confluency.

To prepare samples for dSTORM, cells were first washed twice with pre-warmed phosphate-buffered saline (PBS). Cells were fixed with 4% paraformaldehyde (PFA) in PBS for 15 minutes at room temperature, followed by blocked with a blocking buffer (5% Bovine Serum Albumin [BSA] in PBS) for 30 minutes at room temperature.

Samples were incubated with a primary monoclonal anti-PTK7 antibody [Novus Biologicals, LLC., 5 μg/mL] in blocking buffer overnight at 4 °C. After three washes with PBS, cells were incubated with a secondary antibody conjugated to Alexa Fluor 647 (Goat anti-Mouse IgG, Alexa Fluor 647, Invitrogen) at a 1:200 dilution in blocking buffer for 1 hour at room temperature in the dark. Finally, the samples were washed three times with PBS and stored in PBS at 4°C until imaging.

20 μL of imaging buffer was dropped on a large slide (24 mm x 50 mm); the imaging buffer contained Tris (50 mM,pH 8.0), NaCl (10 mM), glucose (10% w/v), glucose oxidase (500 μg/mL, sigma), catalase (40 μg/mL, sigma), and β-mercaptoethanol (βME) (1% v/v, sigma). Small coverslip (22 mm x 22 mm) containing cells were slowly poured onto larger slides and sealed with nail polish. By inverting the coverslip, observe the apical membrane and basolateral membrane separately.

Super-resolution dSTORM imaging was performed on a Nikon N-STORM system built on an inverted Nikon Eclipse Ti2-E microscope equipped with a perfect focus system (PFS). The system was fitted with a 100× oil-immersion objective lens (CFI Apochromat TIRF 100x, NA 1.49). Fluorophores were excited using a 647 nm laser line, and a 405 nm laser was used for photo-reactivation of the Alexa Fluor 647 dye.

Imaging was conducted in a photoswitching buffer. For each region of interest, a continuous sequence of 15,000 raw frames was acquired using an sCMOS camera (Hamamatsu ORCA-Flash4.0 V3) at a frame rate of approximately 33 Hz (30 ms exposure time per frame). The laser power and camera gain were adjusted to ensure a high density of single-molecule blinking events with a high signal-to-noise ratio.

The raw STORM image stacks were processed using Nikon's NIS-Elements AR software with the N-STORM analysis module. Single-molecule localization and fitting were performed using a 2D Gaussian model to determine the precise coordinates of each detected fluorophore. The resulting localization list was used to generate a super-resolved, reconstructed image. Drift correction was applied during the reconstruction process based on fiducial markers or image correlation.

Quantitative analysis of PTK7 clustering was performed on the reconstructed super-resolution images using ImageJ software. For each image, a region of interest (ROI) was selected on the cell membrane. The reconstructed STORM images were converted to an 8-bit grayscale format, and a uniform intensity threshold was applied across all images to segment the PTK7 clusters from the background. The "Analyze Particles" function was then used to automatically measure the area, density, and number of individual PTK7 clusters within the ROI. The parameters for "Analyze Particles" were set to exclude single, non-clustered localizations and noise (e.g., by setting a minimum particle area of 0.01 µm^2^). The resulting data were exported for statistical analysis in GraphPad Prism 9.0.

**The Expression of PTK7 Determined by Flow Cytometry**

PTK7 expression on the cell surface was analyzed by flow cytometry. hTERT-HPNE, MIAPaCa-2, PANC-1, and PaTu-8988T cells were incubated with either Alexa Fluor 488-labeled IgG or Alexa Fluor 488-labeled anti-PTK7 antibody (Novus Biologicals, LLC) for 1 hour at 4°C. After incubation, cells were washed with 1 mL DPBS, resuspended in 200 μL DPBS, and analyzed using a BD FACSVerse flow cytometer. Fluorescence intensity was quantified with FlowJo v10.9. Experiments were performed in triplicate.

**Cell Proliferation Assay**

Cell viability and proliferation kinetics were evaluated using the Cell Counting Kit-8 (CCK-8; A311, Vazyme, China). For the assay, cells were seeded at a density of 3,000 cells per well in 96-well microplates and allowed to adhere during a 24-hour incubation under standard culture conditions (37°C, 5% CO₂). Following this attachment period, cells were treated with or without TGF-β (10 ng/mL) in fresh culture medium. Viability measurements were performed daily over a 72-hour time course post-treatment. At each time point (Days 1, 2, and 3), 10 μL of CCK-8 reagent was added to each well according to the manufacturer's protocol. Plates were subsequently incubated for 2 hours (37°C, protected from light) to allow for the enzymatic conversion of WST-8 to water-soluble formazan dye by cellular dehydrogenases. The absorbance of formazan was quantified at 450 nm using a Synergy 2 microplate reader (BioTek, USA), with background subtraction using blank wells containing medium and CCK-8 reagent without cells. Proliferation curves were constructed by plotting the mean absorbance values (± SD) against time to visualize treatment-dependent effects on cell growth dynamics.

**Cell Cycle Assay**

Cell cycle was analyzed with the Cell Cycle and Apoptosis Analysis Kit (C1052, Beyotime, China) following the manufacturer’s protocol. hTERT-HPNE, MIAPaCa-2, PANC-1, and PATU8988T cells were treated with or without TGF-β (10 ng/mL) for 48 hours. Cell cycle distribution was determined by flow cytometry and analyzed using FlowJo v10.9. All experiments were performed in triplicate.

**Cell Apoptosis Assay**

Cell apoptosis was assessed using the Annexin V-FITC Apoptosis Detection Kit (C1062L, Beyotime, China) following the manufacturer’s instructions. hTERT-HPNE, MIAPaCa-2, PANC-1, and PATU8988T cells were treated with or without TGF-β (10 ng/mL) for 48 hours. Apoptosis rates were determined by flow cytometry and analyzed with FlowJo v10.9. Experiments were performed in triplicate.

**Wound Healing Assay**

Cell migration was assessed using hTERT-HPNE, MIA PaCa-2, PANC-1, PaTu-8988t, MCF10A, MCF-7, MDA-MB-231, NCM460, HCT116, and SW620 cell lines and PAAD-23020, -22001, -21010 primary pancreatic cancer cells from three clinical patients. Cells were seeded in wells of a 6-well plate and grown to 90% confluence. A sterile 10 µL pipette tip was used to create a linear scratch in the cell monolayer. After wounding, cells were washed with PBS and cultured in fresh medium. Images of the wound area were captured at 0 and 24 hours using a microscope. Cell migration was quantified using ImageJ, and the wound closure rate was calculated. Each experiment was performed in triplicate (n=3), and data were analyzed using one-way ANOVA to assess statistical significance.


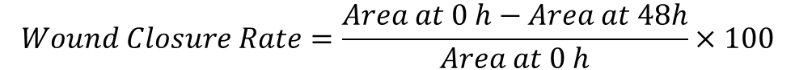


**Transwell Migration Assay**

To evaluate cellular migration, transwell assays were conducted utilizing 8.0 µm pore-sized PET membrane inserts from BD Falcon in wells of a 24-well plate. hTERT-HPNE, MIA PaCa-2, PANC-1 and PaTu-8988t cell lines were cultured for 24 hours. Subsequently, 30,000 cells suspended in 200 μL of serum-free medium were placed in the upper chamber of the transwell, while the lower chamber of the 24-well plate received 600 μL of complete growth medium. Following a 48-hour incubation, the cells that had migrated to the lower chamber were fixed with 4% paraformaldehyde, stained with 0.2% crystal violet, and quantified by counting on representative microscopic images. The experiment was repeated three times independently.

**TGF-β Treatment for EMT Process**

hTERT-HPNE, MIA PaCa-2, PANC-1, and PaTu-8988t cells were plated at 70%–80% confluence and allowed to rest in 1% FBS-containing DMEM for 24 h. These cell lines were treated with 10 ng/mL of recombinant human TGF-β1 for 48 h. After EMT, cell lines were stained with antibodies of EMT key markers, using a confocal fluorescence microscope.

**EMT Marker Analysis**

The downregulation of E-cadherin and the upregulation of vimentin are pivotal events in the process of Epithelial-Mesenchymal Transition (EMT). Alexa Fluor 488-conjuged E-cadherin antibody and Alexa Fluor 594-conjuged vimentin antibody were used to detect the expression of E-cadherin and vimentin in living cells after TGF-β treatment. PANC-1, MIA PACA-2, and PATU-8988T cells were cultured on chamber slides to appropriate confluence and treated with 10 ng/mL TGF-β for 48 hours. Cells were then fixed with 4% paraformaldehyde for 15 minutes, permeabilized with 0.2% Triton X-100 (P0096, Beyotime, China) for 10 minutes, and blocked with 10% BSA for 1 hour. Primary antibodies were applied and incubated overnight at 4°C. After washing with PBS and staining with DAPI for 10 minutes, images were captured using a confocal fluorescence microscope.

Antibodies used: Alexa Fluor 488-conjugated E-cadherin (3199s, CST, USA, 1:100) and Alexa Fluor 594-conjugated vimentin (7675, CST, USA, 1:100).

**PTK7 Antibody Inhibition Assay**

For single-molecule tracking, PaTu-8988t and primary patient cells PAAD-21010 were first co-incubated with an anti-PTK7 monoclonal antibody (1:200 dilution) and the Sgc8c-Atto647N aptamer in binding buffer for 30 minutes at 4 °C. Following incubation, unbound antibody and aptamer were removed by washing the cells three times with washing buffer.

For the wound healing assay, scratches were made on confluent monolayers of PaTu-8988t and PAAD-21010 cells, which were then cultured for 24 hours in the continuous presence of an anti-PTK7 monoclonal antibody (1:200 dilution)

**Western Blot Assay**

Cells were lysed in RIPA buffer containing protease and phosphatase inhibitors. Protein concentrations were quantified using the BCA Protein Assay Kit (P0009, Beyotime, China) and normalized. Equal amounts of protein (20 μg) were separated on a 10% SDS-PAGE gel and transferred to PVDF membranes. Membranes were blocked with 5% skimmed milk for 2 hours at room temperature and incubated overnight at 4 °C with primary antibodies. The next day, membranes were incubated with HRP-conjugated secondary antibodies for 1 hour at room temperature. Protein bands were detected using a ChemiDoc Imaging System (Bio-Rad, USA).

The antibodies used in this study included GAPDH (A19056, ABclonal, China, 1:1000) and HRP-conjugated secondary antibody (AS014, ABclonal, China, 1:5000).

**The Molecular Docking Between PTK7 and ROR2**

The domain of human ROR2 (UniProt: Q01974) was modeled using the AlphaFold2-predicted structure (AF-Q01974-F1). The structure of human PTK7 (UniProt: Q13308) was derived from the AlphaFold-predicted structure (AF-Q13308-F1). Global protein-protein docking was performed using the HDOCK server (v2.0) with default parameters. The HDOCK global docking using ITScorePP scoring yielded a top-ranked complex with a docking score of -162. The resulting confidence score (0.82) exceeded the 0.7 threshold, strongly supporting the biological plausibility of the predicted binding mode and justifying subsequent interaction analysis.

**Immunoprecipitation Assay**

Cell lysates were prepared, and the supernatant was collected on ice. The lysates were incubated overnight with 2 μg of antibody at 4°C to form immune complexes. Magnetic beads (50 μL) were pre-washed with washing buffer and then incubated with the immune complexes at room temperature for 3 hours on a vertical rotator. After extensive washing to remove nonspecific binders, the beads were resuspended in 30–50 μL of 2× SDS-PAGE Sample Loading Buffer and heated at 95°C for 10 minutes to elute the bound proteins. SDS-PAGE analyzed the eluted proteins.

The following antibodies were used for Co-IP and subsequent analysis: SAPK/JNK (E7R5D) (#67096S, Cell Signaling), Phospho-SAPK/JNK (T183/Y185) (98F2) (#4671T, Cell Signaling), PTK7/CCK4 (D2Z1N) (#25618S, Cell Signaling), ROR2 (D3B6F) (#88639S, Cell Signaling), Phospho-ROR2 (Ser449) (PA564808, Invitrogen), Dishevelled 2 (AB228804, Abcam), Dishevelled 2 (EPNCIR145) (phospho S143) (AB124933, Abcam), and Rabbit Control IgG (#AC005, ABclonal).

**siRNA-Mediated ROR2 Silencing**

PATU-8988t cells were plated in 6-well culture plates at approximately 40–50% confluence and incubated overnight prior to siRNA transfection. Gene knockdown was performed by introducing 50 nM of ROR2-specific siRNA or a non-targeting control siRNA using Lipofectamine 2000 (11668019, Thermo Fisher Scientific, USA) according to the manufacturer’s instructions.

48 hours after transfection, the reduction of ROR2 expression was examined by Western blot. Following confirmation of knockdown efficiency, alterations in cell migratory behavior were evaluated using wound healing and Transwell migration assays.

Sequences used in the study were as follows:
siROR2-1: sense 5′-CUAGUGUACGACAAGCUGA(dT)(dT)-3′; antisense 5′-UCAGCUUGUCGUACACUAG(dT)(dT)-3′.
siROR2-2: sense 5′-GACAGAAUAUGGUUCACGA(dT)(dT)-3′; antisense 5′-UCGUGAACCAUAUUCUGUC(dT)(dT)-3′.

siROR2-3: sense 5′-GAUUACAGAGGAACGGCAA(dT)(dT)-3′; antisense 5′-UUGCCGUUCCUCUGUAAUC(dT)(dT)-3′.
siNC: sense 5′-UUCUCCGAACGUGUCACGU(dT)(dT)-3′; antisense 5′-ACGUGACACGUUCGGAGAA(dT)(dT)-3′.

**Wnt5α Binding with PTK7 and ROR2**

MIA PaCa-2, PANC-1, and PaTu-8988t cells, with or without TGF-β treatment (10 ng/mL, 48 h), were digested and incubated with FITC-conjugated recombinant Wnt5α protein (100 ng/mL) at 4°C for 1 hour. FITC fluorescence signal was subsequently analyzed using flow cytometry.

**qRT-PCR**

Total RNA was isolated from samples using the RNA Extraction Kit for Animals (RK30120, ABclonal, China) following the manufacturer's protocol. Complementary DNA (cDNA) was synthesized using the RT Master Mix for qPCR (RK20428, ABclonal, China). Quantitative real-time PCR (qRT-PCR) was conducted with 2X Universal SYBR Green Fast qPCR Mix (RK21203, ABclonal, China) on a QuantStudio 5Dx system (Applied Biosystems). Relative gene expression levels were determined using the 2^-ΔΔCt^ method with GAPDH serving as the reference gene. To confirm amplification specificity, melting curve analysis was performed at the conclusion of the PCR cycles. The primer sequences utilized in this study are provided below:

| Gene Name | Forward Primer (5'-3') | Reverse Primer (5'-3') |
| --- | --- | --- |
| FOS | CCGGGGATAGCCTCTCTTACT | CCAGGTCCGTGCAGAAGTC |
| MMP1 | AAAATTACACGCCAGATTTGCC | GGTGTGACATTACTCCAGAGTTG |
| BCL2 | GGTGGGGTCATGTGTGTGG | CGGTTCAGGTACTCAGTCATCC |
| MMP9 | TGTACCGCTATGGTTACACTCG | GGCAGGGACAGTTGCTTCT |
| NOS2 | TTCAGTATCACAACCTCAGCAAG | TGGACCTGCAAGTTAAAATCCC |
| COL1A1 | GAGGGCCAAGACGAAGACATC | CAGATCACGTCATCGCACAAC |
| IL6 | ACTCACCTCTTCAGAACGAATTG | CCATCTTTGGAAGGTTCAGGTTG |
| GAPDH | GGAGCGAGATCCCTCCAAAAT | GGCTGTTGTCATACTTCTCATGG |

**Immunohistochemistry**

Tumor tissues were fixed with 10% neutral buffered formalin, encapsulated in paraffin, and cut into sections measuring 4–5 μm in thickness. These sections were then deparaffinized, rehydrated, and submitted to antigen retrieval. To block endogenous peroxidase activity, a specific protocol was followed, after which the sections were incubated with primary antibodies targeting PTK7. Post-washing, the sections were treated with secondary antibodies, processed with streptavidin-HRP, and finally developed with the DAB substrate to produce a colored reaction. The sections were subsequently scanned using the Digital Pathology Slide Scanner (KFBIO KF-PRO-120, China).

**Supplementary Figures**


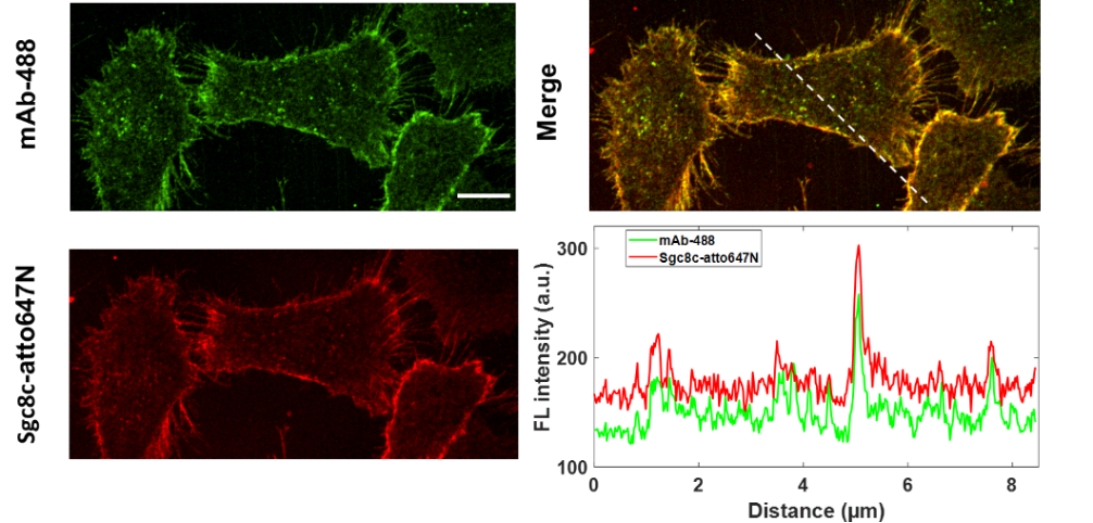


**Figure S1.** **Specific binding of Sgc8c-Atto674N to PTK7.** Colocalization of PTK7 and Sgc8c in PaTu-8988t cells labeled with PTK7 antibody and Atto647N, respectively. Fluorescence intensity of aptamer and antibody along dashed line was shown. (mAb-488: green channel, Sgc8c-Atto647N: red channel, merge: yellow channel) Scale bar: 10 µm.


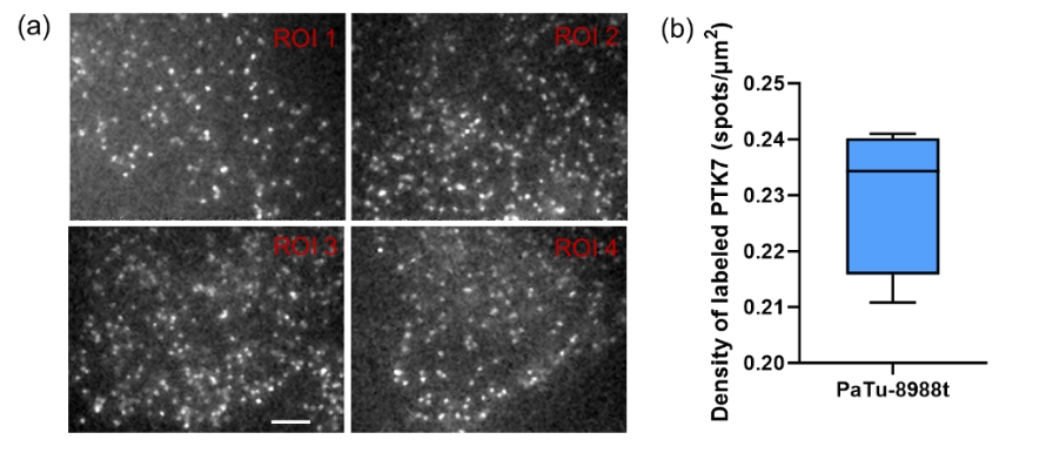


**Figure S2. Quantitative analysis of PTK7 cell surface label density.** (a) PTK7 fluorescent spots in Patu-8988t cells from different images. (b) Statistical analysis of the number of PTK7 fluorescent spots on the cell surface per unit area. Scale bar: 5 µm.


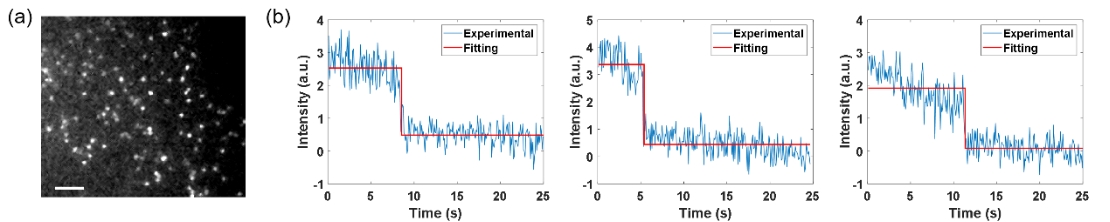
**Figure S3. Single-molecule photobleaching.** (a) TIRFM image of single PTK7 molecules spotted in Patu-8988t cells. (b) Representative intensity profiles (blue) of PTK7 receptors labeled with Sgc8c-Atto647N. Intensity profiles were fitted with a step-fitting algorithm (red). Scale bar: 5 µm. Note: This panel Figure S3(a) displays the same source image presented in Figure S2(a) ROI 1, but here it is used to demonstrate the single molecule events.


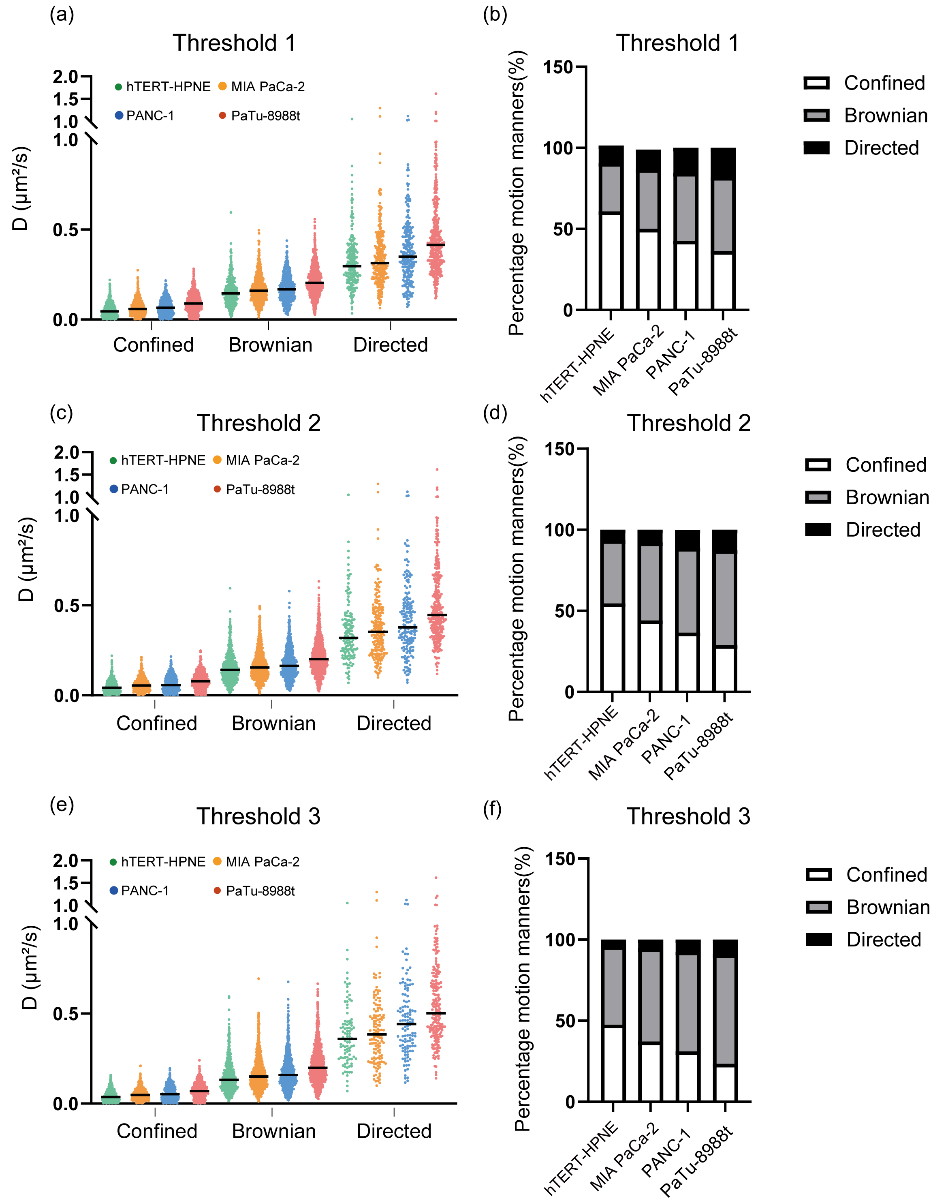


**Figure S4. Classification of PTK7 diffusion dynamics under varying anomalous diffusion exponent (α) thresholds.** Diffusion coefficient (D) and percentage of motion modes across three thresholds (Threshold 1, 2, and 3, corresponding to (a)-(b), (c)-(d), and (e)-(f), respectively) in hTERT-HPNE, MIA PaCa-2, PANC-1, and PaTu-8988t cells. Threshold 1: Confined (α < 0.85), Brownian (0.85 < α < 1.15), Directed (α > 1.15); Threshold 2: Confined (α< 0.8), Brownian (0.8< α <1.2), Directed (α > 1.2); Threshold 3: Confined (α < 0.75), Brownian (0.75< α < 1.25), Directed (α > 1.25)


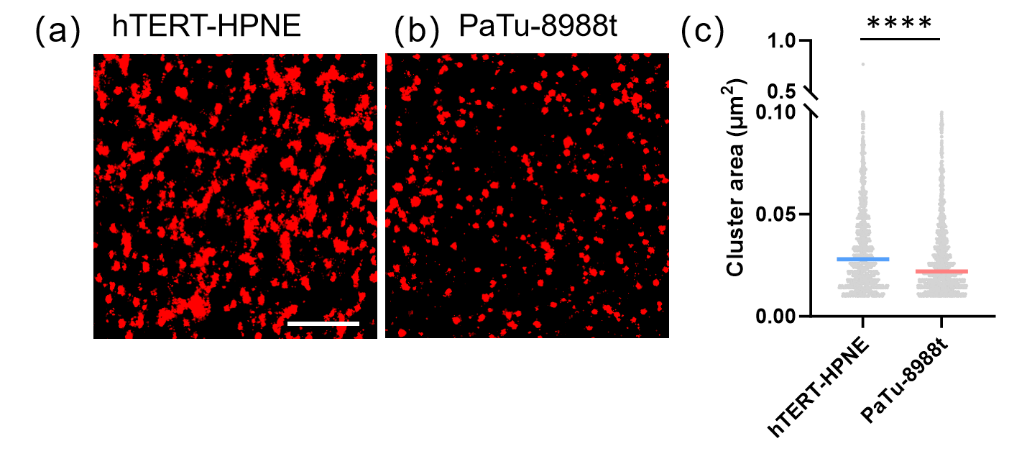


**Figure S5. STORM imaging of PTK7 on basal membrane.** Reconstructed STORM images of PTK7 on the hTERT-HPNE(a) and PaTu-8988t(b). (c) Distribution of cluster area and median values (marked with blue and red lines) in hTERT-HPNE and PaTu-8988t cells. 15000 raw frames were acquired with an sCMOS camera (Hamamatsu FLASH4.0) with 30 ms exposure time. Significance levels: ns, p > 0.05, * p < 0.05, ** p < 0.01; *** p < 0.001, **** p < 0.0001 by unpaired Student’s t test.


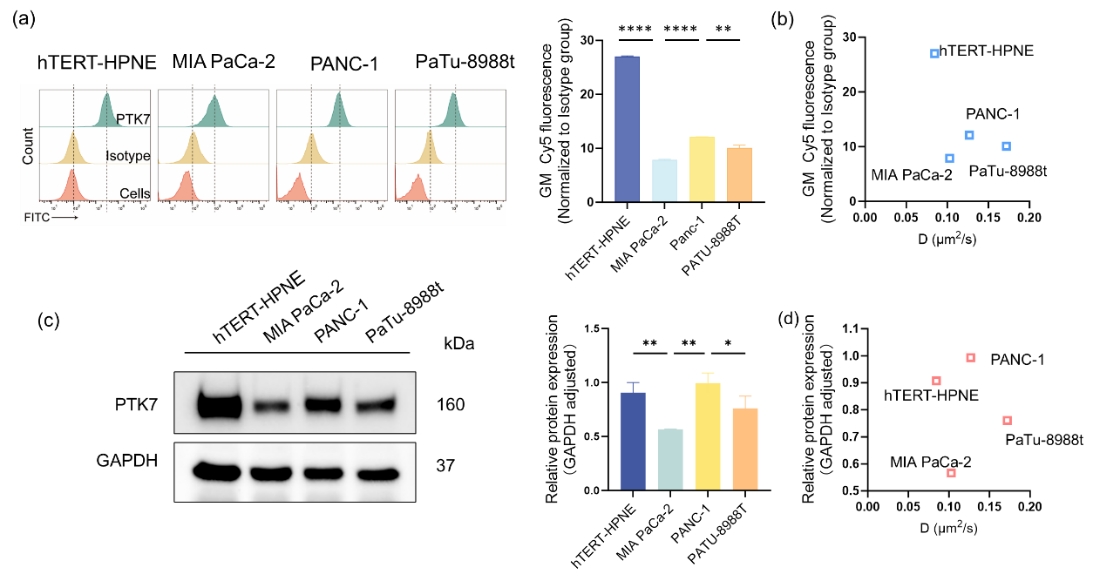
**Figure S6. PTK7 level in pancreatic cell lines.** (a) Flow cytometry to monitor PTK7 level on the cell membrane. (b) Scatter plot showing the relationship between diffusion coefficients (D) and PTK7 level on the cell membrane, indicating no significant correlation. (c) PTK7 level, as determined by Western blot (left), and the corresponding quantification (right). (d) Scatter plot showing the relationship between diffusion coefficients (D) and total PTK7 level, indicating no significant correlation. All data are expressed as mean ± SD (n = 3 biological replicates per group from three independent experiments). Significance levels: ns (not significant), p > 0.05; *p < 0.05; **p < 0.01; ***p < 0.001; ****p < 0.0001 by (one-way ANOVA with Tukey's multiple comparisons test).


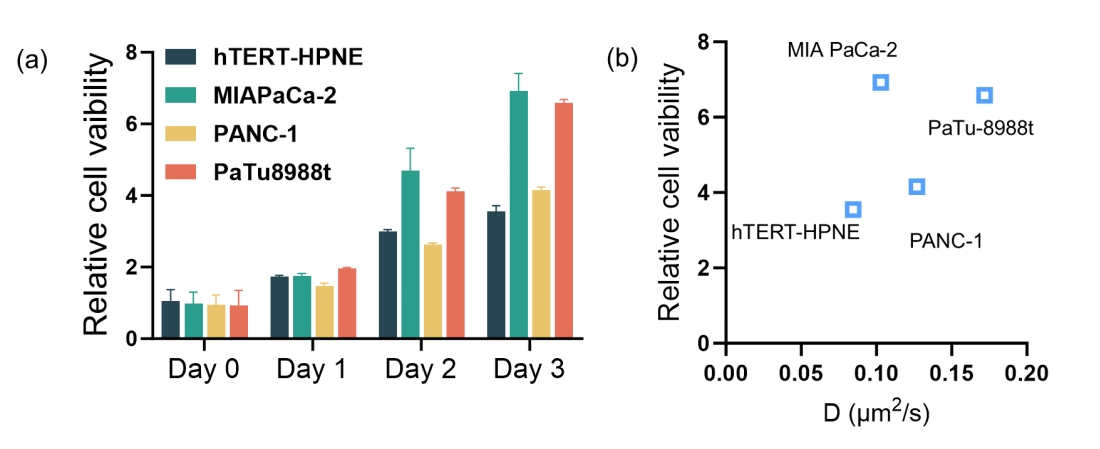
**Figure S7. Cell proliferation in pancreatic cell lines.** (a) Cell growth measured by CCK-8 kit among hTERT-HPNE, MIA PaCa-2, PANC-1, and Patu-8988t cell lines. (b) Scatter plot showing the relationship between diffusion coefficients (D) and proliferative capacity, indicating no significant correlation. All data are expressed as mean ± SD (n = 3 biological replicates per group from three independent experiments).


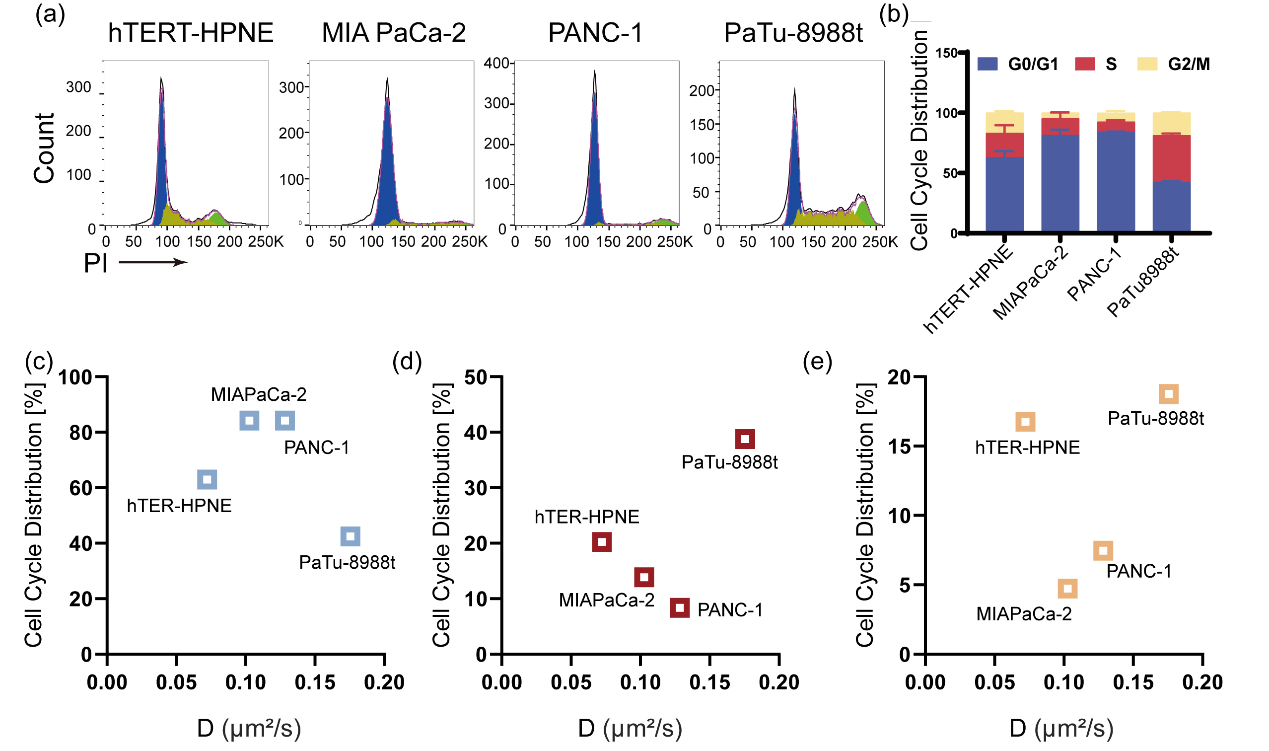


**Figure S8. Cell cycle of pancreatic cell lines.** (a) Cell cycle distribution analysis of hTERT-HPNE, MIA PaCa-2, PANC-1, and Patu-8988T cell lines using propidium iodide (PI) staining. (b) Statistical summary of cell cycle phases in Figure S6a. All data are expressed as mean ± SD (n = 3 biological replicates per group from three independent experiments).


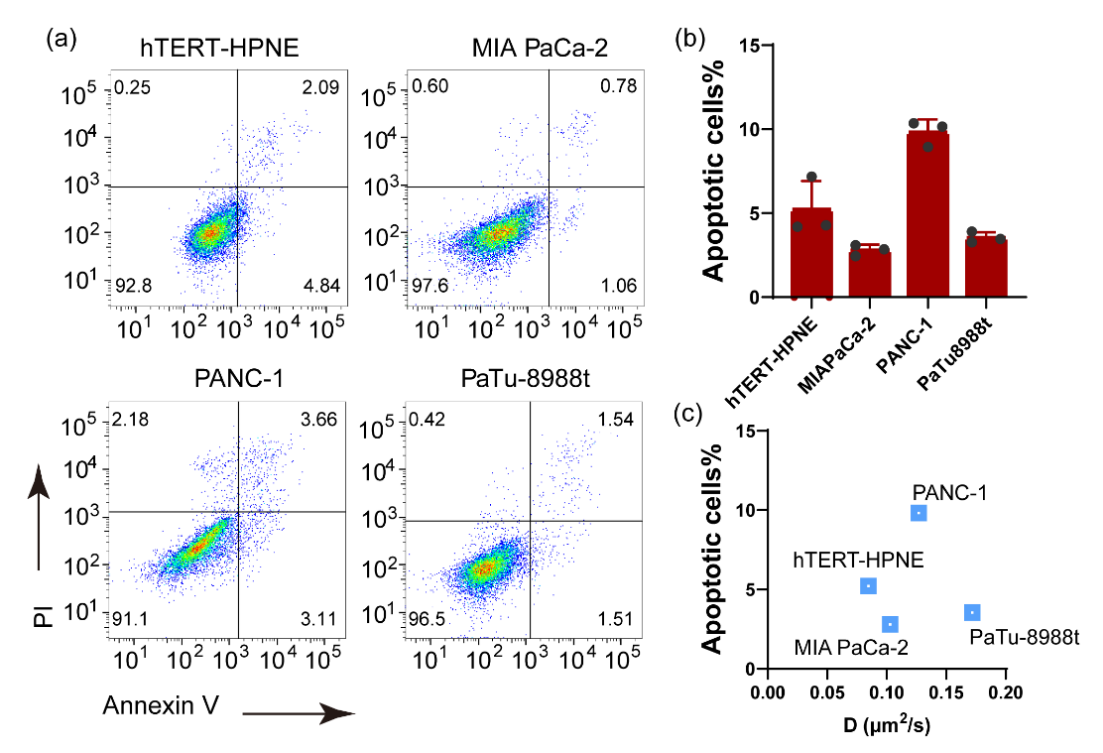


**Figure S9. Cell apoptosis analysis in pancreatic cell lines.** (a)-(b) Cell apoptosis measured by apoptosis detection kit among hTERT-HPNE, MIA PaCa-2, PANC-1, and Patu-8988t cell lines. (c) Scatter plot illustrating the relationship between diffusion coefficients (D) and cell apoptosis, showing no significant correlation. All data are expressed as mean ± SD (n = 3 biological replicates per group from three independent experiments).


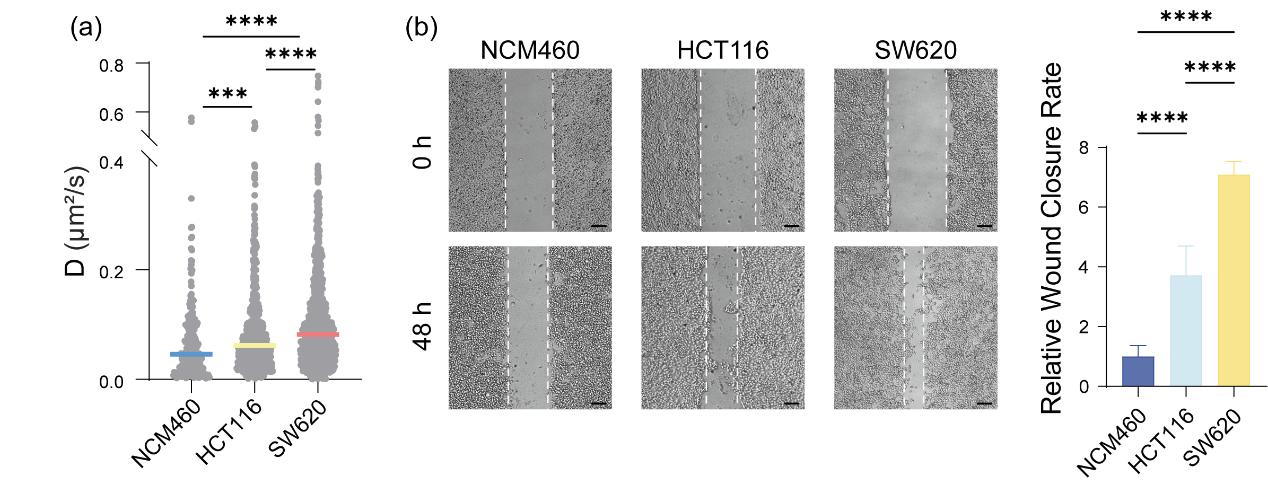


**Figure S10. PTK7 dynamics and colorectal cell migration.** (a) Distribution and median values of diffusion coefficients (D) of PTK7 molecules in colorectal (NCM460, HCT116, SW620) cell lines. (b) Wound healing of colorectal cell lines. Scale bar: 200 µm. All data are expressed as mean ± SD (n = 3 biological replicates per group from three independent experiments). Significance levels: ns (not significant), p > 0.05; *p < 0.05; **p < 0.01; ***p < 0.001; ****p < 0.0001 (one-way ANOVA with Tukey's multiple comparisons test).


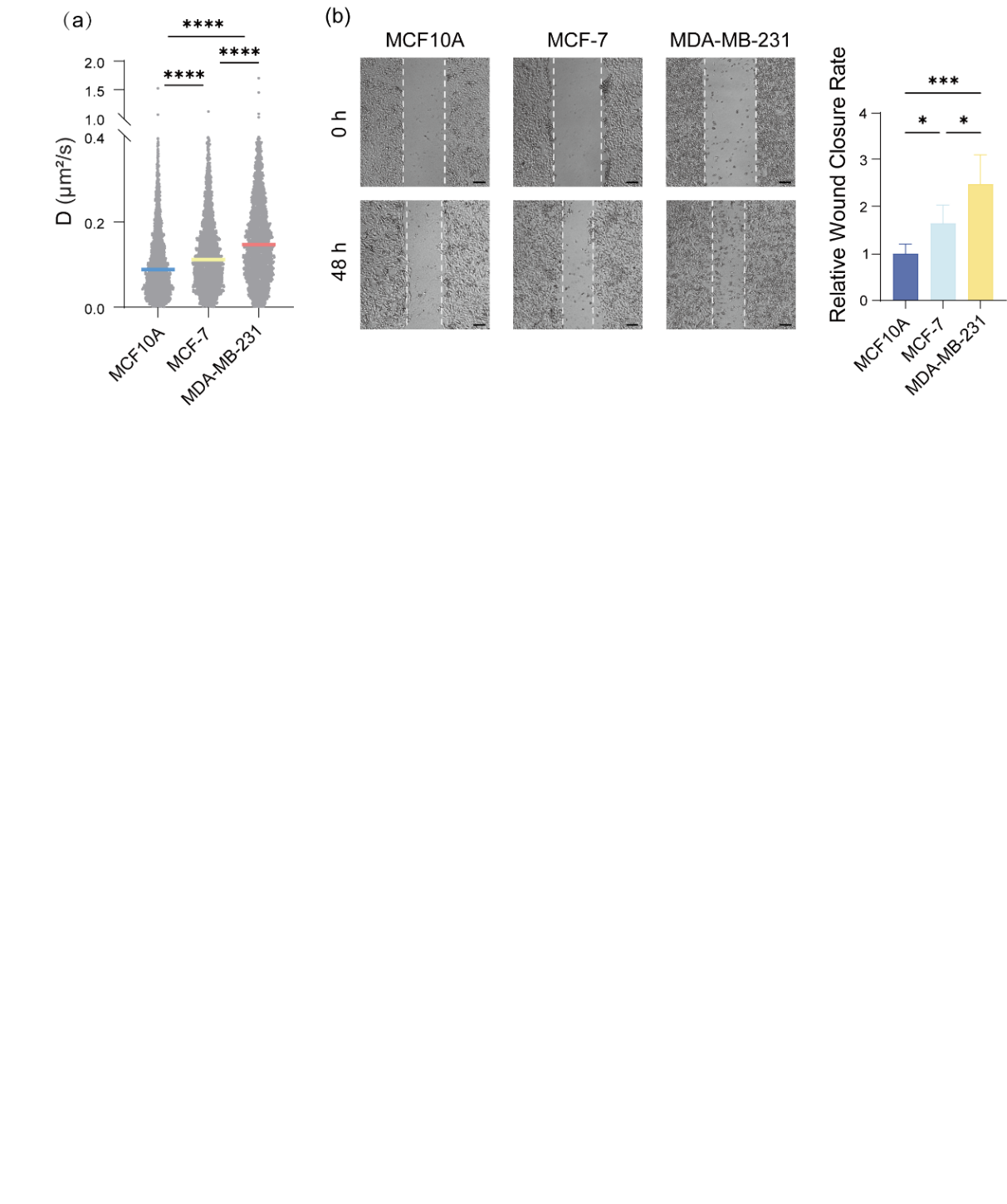


**Figure S11. PTK7 dynamics and mammary cell migration.** (a) Distribution and median values of diffusion coefficients (D) of PTK7 molecules in mammary (MCF10A, MCF-7, MDA-MB-231) cell lines. (b) Wound healing of colorectal cell lines. Scale bar: 200 µm. All data are expressed as mean ± SD (n = 3 biological replicates per group from three independent experiments). Significance levels: ns (not significant), p > 0.05; *p < 0.05; **p < 0.01; ***p < 0.001; ****p < 0.0001 by one-way ANOVA with Tukey's multiple comparisons test.


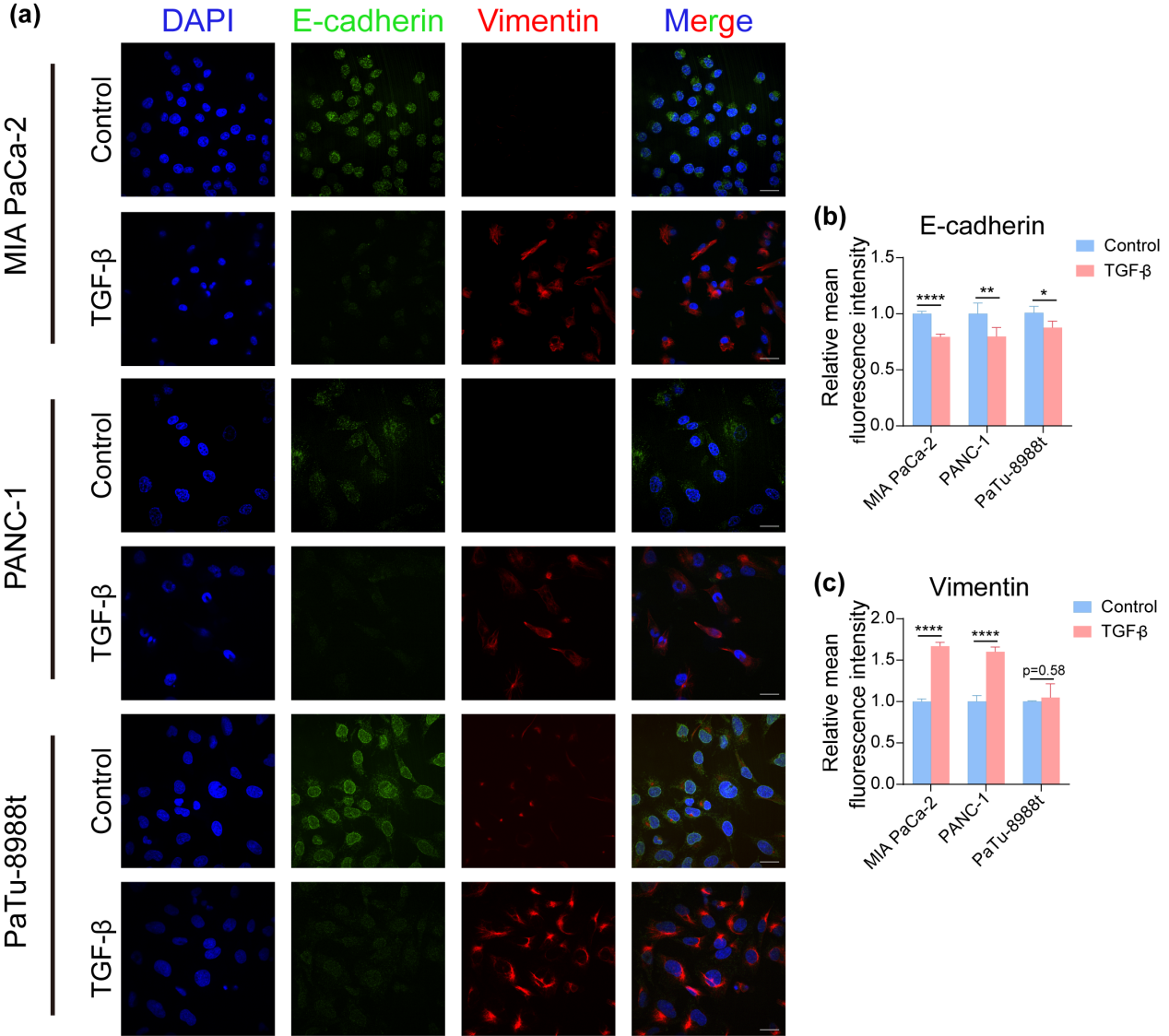


**Figure S12. Detection of EMT marker expression.** (a) Immunofluorescence staining of E-cadherin and vimentin expression before and after EMT in MIA PaCa-2, PANC-1, and PaTu-8988t cell lines. E-cadherin and vimentin were stained with Alexa Fluor488-labed antibody (green) and Alexa Fluor594-labeded antibody (red) respectively. Cell nuclei were stained with Hoechst 33342 (blue). (Scale bar: 20 µm). (b)-(c) Quantification of E-cadherin and vimentin expression level by flow cytometry assay. All data are expressed as mean ± SD (n = 3 biological replicates per group from three independent experiments). Significance levels: ns, p > 0.05, * p < 0.05, ** p < 0.01; *** p < 0.001, **** p < 0.0001 by unpaired two-tailed Student’s t test.

**
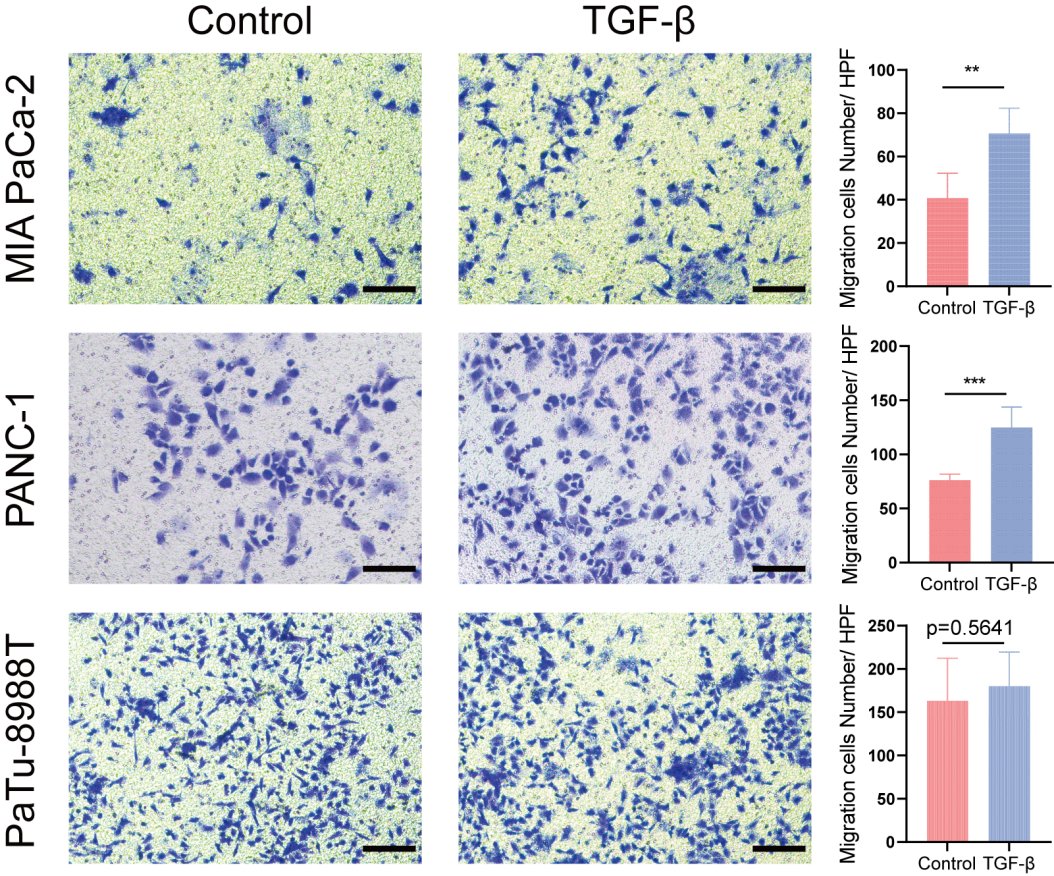
**

**Figure S13.** **Cell migration treated with TGF-β.** Cell migration ability of three cell lines before and after TGF-β treatment (left) and corresponding statistical analysis of migrated cell numbers (right). Scale bar: 100 µm. All data are expressed as mean ± SD (n = 3 biological replicates per group from three independent experiments). Significance levels: ns, p > 0.05, * p < 0.05, ** p < 0.01; *** p < 0.001, **** p < 0.0001 by unpaired two-tailed Student’s t test.


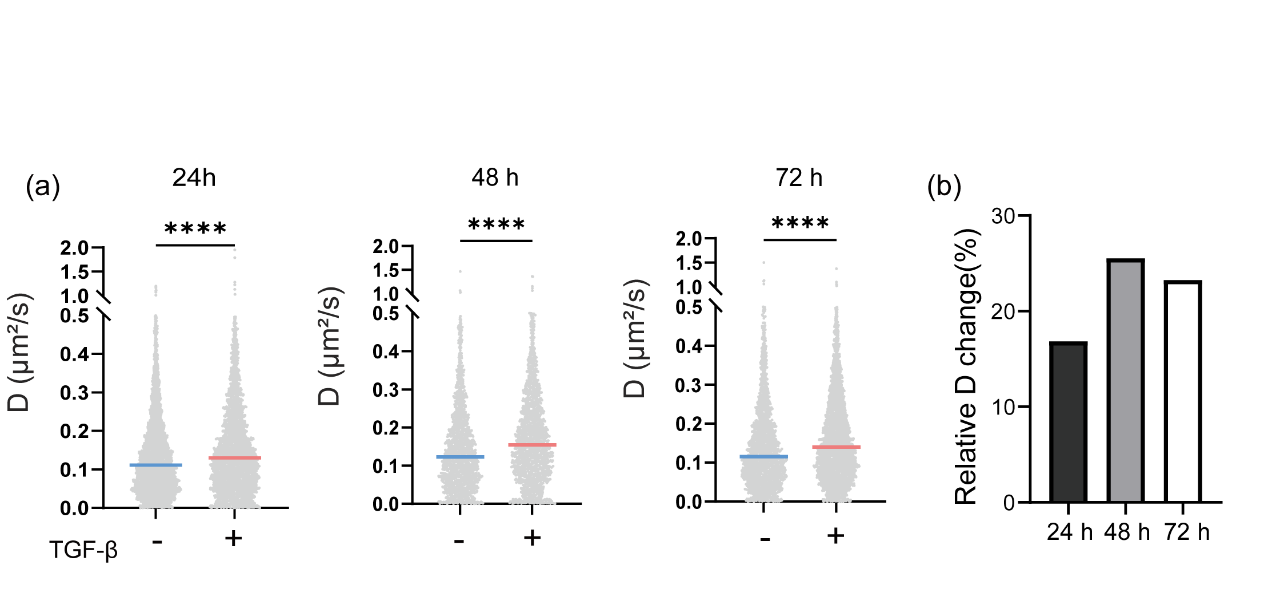


**Figure S14. TGF-β induces time-dependent change in PTK7 diffusion.** (a) Distribution of D values for PTK7 in PANC-1 cells, either untreated (-) or treated (+) with TGF-β for 24, 48, and 72 hours. Median values are marked with blue and red lines. (b) The relative change in the median diffusion coefficient of treated cells normalized to the control. Significance levels: ns, p > 0.05, * p < 0.05, ** p < 0.01; *** p < 0.001, **** p < 0.0001 by unpaired two tailed Student’s t test.


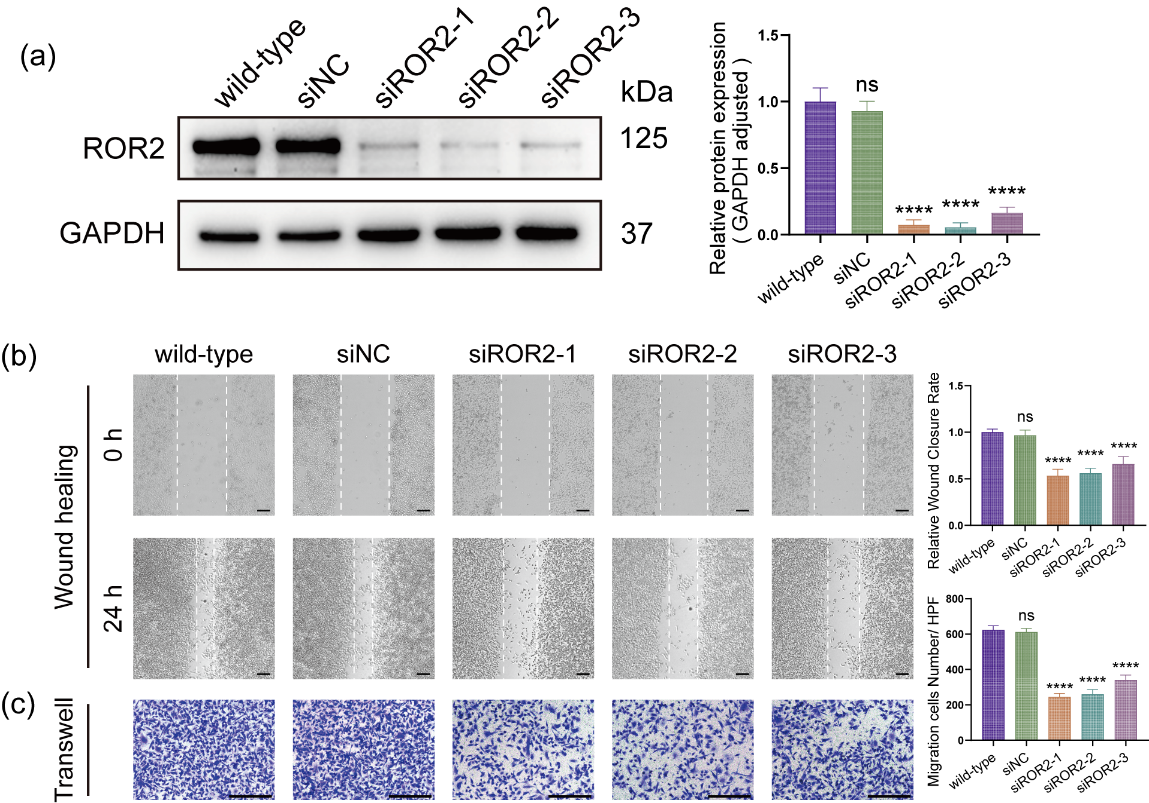


**Figure S15. Effect of ROR2 knockdown on PANC-1 cell motility.** (a) Validation of ROR2 knockdown by Western blot using three different siRNAs. (b) Scratch wound healing assay (Scale bar: 200 µm) and (c) Transwell migration assay (Scale bar: 100 µm) showing reduced cell motility upon ROR2 silencing. All data are expressed as mean ± SD (n = 3 biological replicates per group from three independent experiments). Significance levels: ns (not significant), p > 0.05; *p < 0.05; **p < 0.01; ***p < 0.001; ****p < 0.0001 by (one-way ANOVA with Tukey's multiple comparisons test).


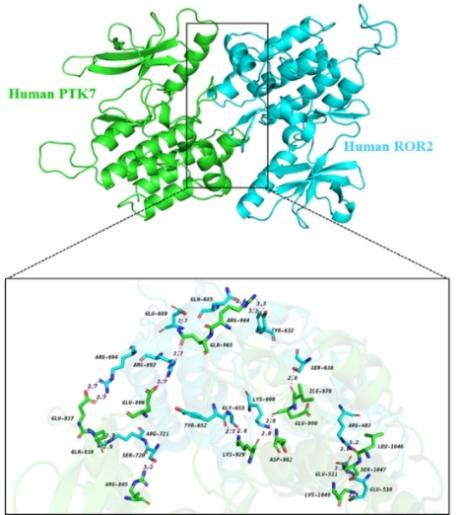


**Figure S16. PTK7 and ROR2 docking prediction.** Computer docking-based PTK7 and ROR2 binding mode simulation.

**
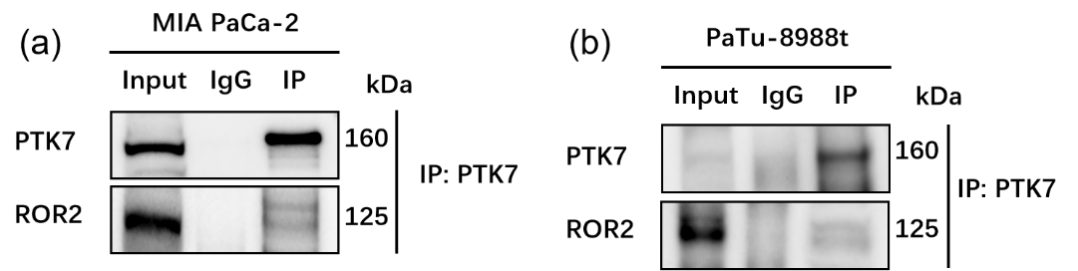
**

**Figure S17. CO-IP assay of PTK7 and ROR2.** Co-immunoprecipitation of PTK7 antibody (or IgG) in MIA PaCa-2 (a) and PaTu-8988t (b) cell lysates, followed by immunoblotting of ROR2 and PTK7.


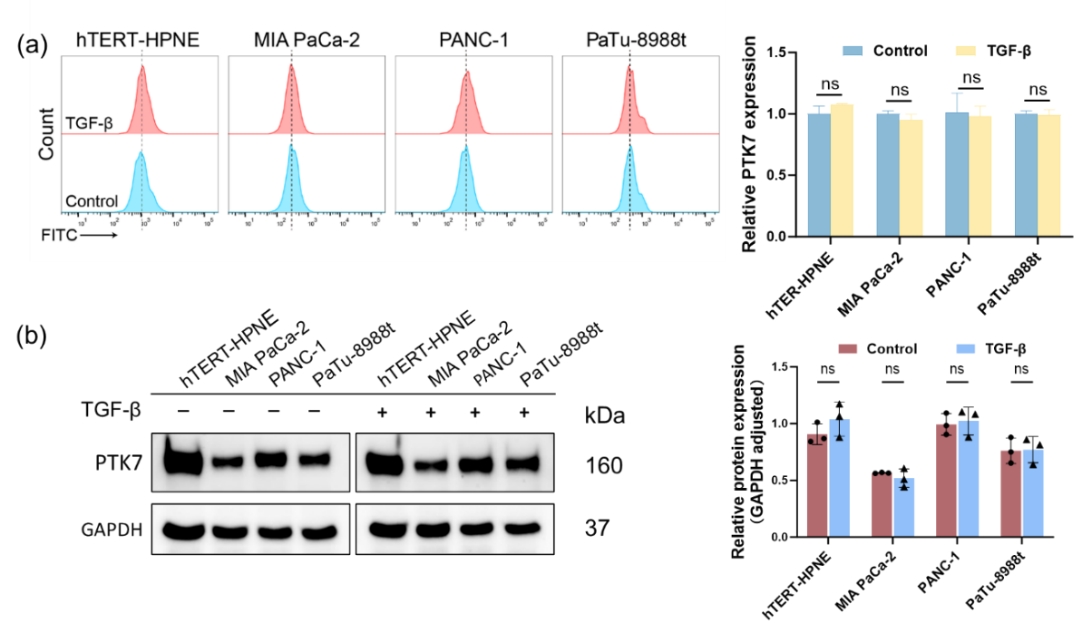


**Figure S18. PTK7 protein expression after TGF-β treatment.** (a)-(b) PTK7 and ROR2 expression level before and after TGF-β treatment, as determined by flow cytometry and Western blot (left) and the corresponding quantification (right) among hTERT-HPNE, MIA PaCa-2, PANC-1, and Patu-8988t cell lines. All data are expressed as mean ± SD (n = 3 biological replicates per group from three independent experiments). Significance levels: ns, p > 0.05, * p < 0.05, ** p < 0.01; *** p < 0.001, **** p < 0.0001 by unpaired two-tailed Student’s t test.

**
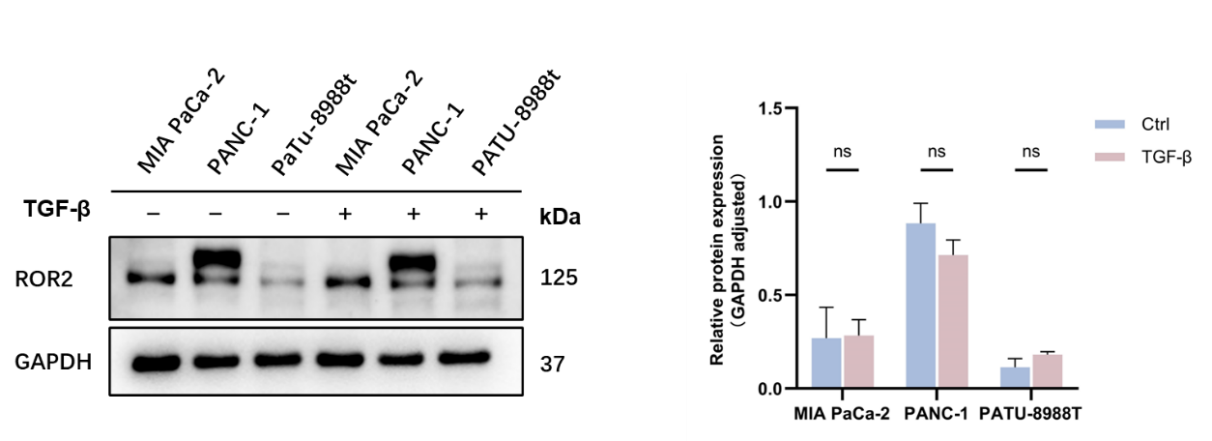
**

**Figure S19. ROR2 protein level after TGF-β treatment.** ROR2 expression level after TGF-β treatment, as determined by Western blot (left) and the corresponding quantification (right) among MIA PaCa-2, PANC-1, and Patu-8988t cell lines. All data are expressed as mean ± SD (n = 3 biological replicates per group from three independent experiments). Significance levels: ns, p > 0.05, * p < 0.05, ** p < 0.01; *** p < 0.001, **** p < 0.0001 by unpaired two-tailed Student’s t test.


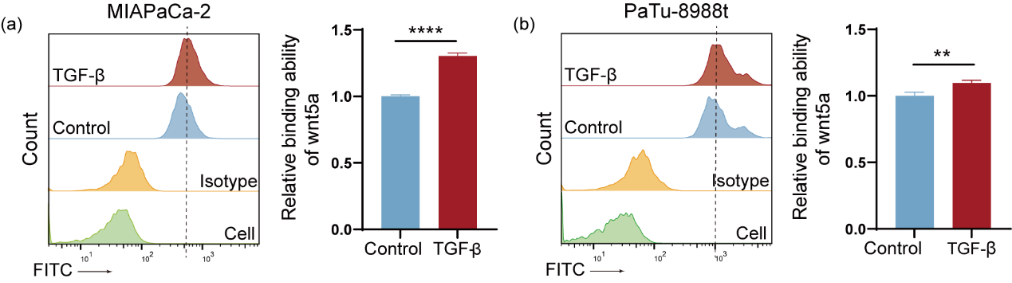


**Figure S20. Binding ability of Wnt5α after TGF-β treatment.** Wnt5α ligand binding with PTK7 and ROR2 in MIA PaCa-2 (a) and PaTu-8988t (b) cells. All data are expressed as mean ± SD (n = 3 biological replicates per group from three independent experiments). Significance levels: ns, p > 0.05, * p < 0.05, ** p < 0.01; *** p < 0.001, **** p < 0.0001 by unpaired two-tailed Student’s t test.


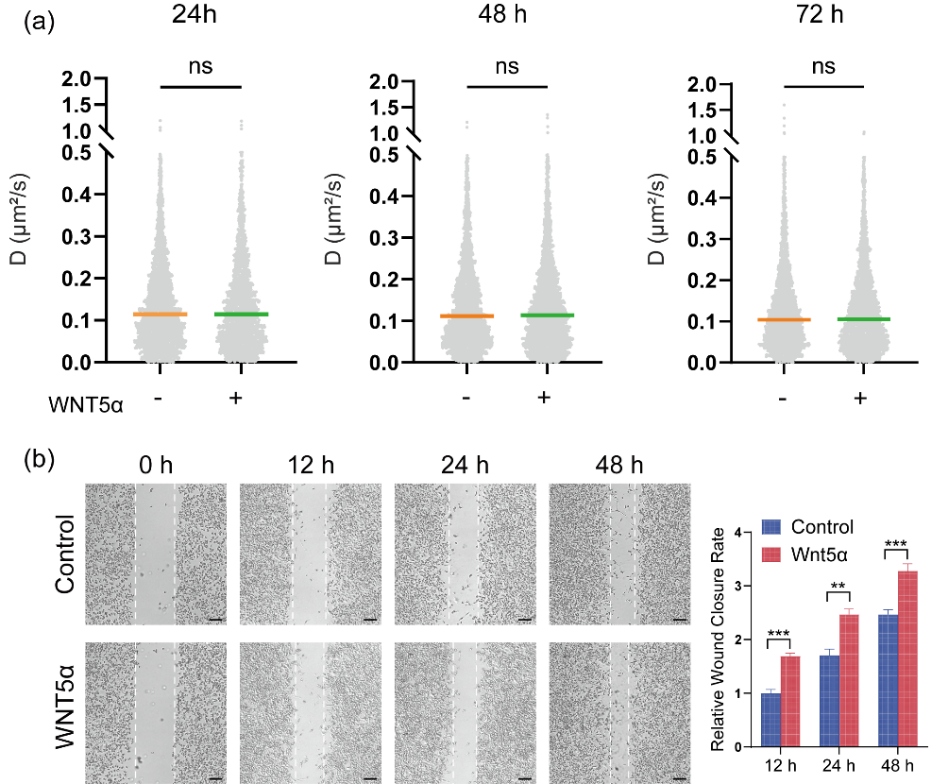


**Figure S21. Effects of Wnt5α on PTK7 diffusion and cell migration.** (a) Diffusion coefficients of PTK7 on the membrane of PANC-1 cells untreated or treated with 100 ng/mL Wnt5α for 24, 48, and 72 h. (b) Representative scratch assay images of PANC-1 cells at 0 h (initial scratch) and after 12, 24, and 48 h of treatment with 100 ng/mL Wnt5a or vehicle control. Scale bar: 200 µm. All data are expressed as mean ± SD (n = 3 biological replicates per group from three independent experiments). Significance levels: ns, p > 0.05, * p < 0.05, ** p < 0.01; *** p < 0.001, **** p < 0.0001 by unpaired two-tailed Student’s t test.

**
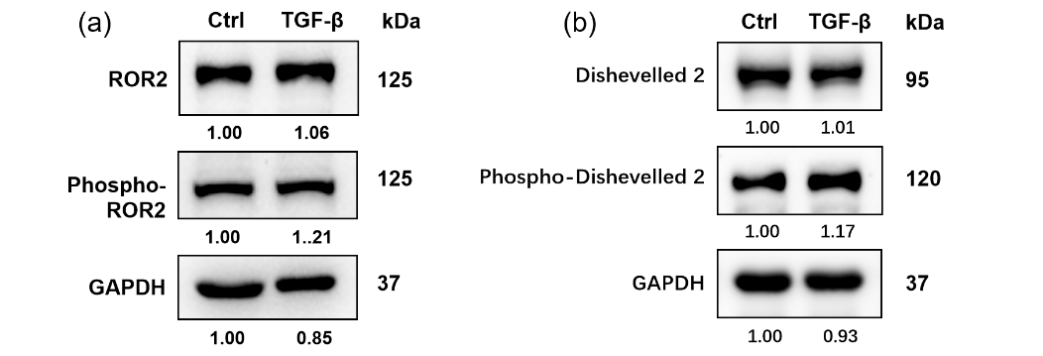
**

**Figure S22. Protein phosphorylation in WNT/PCP pathway.** Phosphorylation level of ROR2 (a) and Dishevelled2 (b) protein with or without TGF-β treatment.


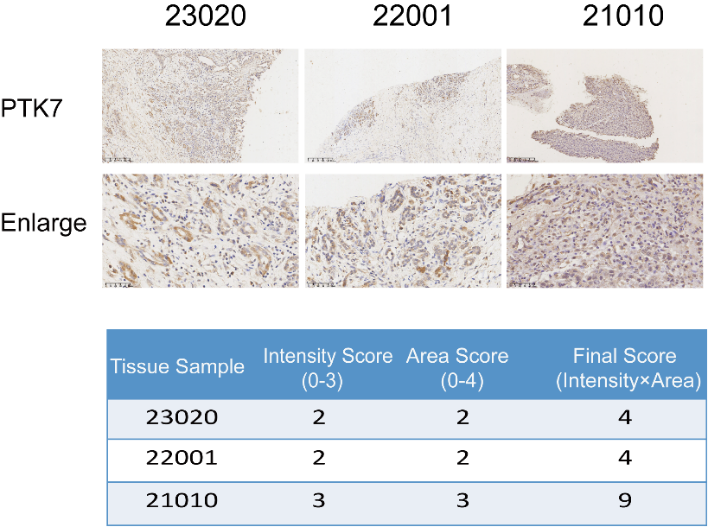


**Figure S23. PTK7 levels in patients’ tissues.** (a) Representative IHC staining images of PTK7 level in three clinical patients, named PAAD-23020, -22001, and -21010 (Scale bar: 200 µm). (b) IHC scoring based on intensity and area.

**Supplementary Movies**

**Movie S1.** Representative single-molecule imaging stream (10 Hz, 10 s) of PTK7 receptors on the surface of PaTu-8988t cells. Image size: 44 × 29 µm².

**Movie S2**. Same imaging stream as Movie S1, with overlaid PTK7 tracks. Random colors distinguish neighboring tracks.

**References**

29. D. Ershov, M.-S. Phan, J. W. Pylvanainen, S. U. Rigaud, L. Le Blanc, A. Charles-Orszag, J. R. W. Conway, R. F. Laine, N. H. Roy, D. Bonazzi, G. Dumenil, G. Jacquemet, J.-Y. “TrackMate 7: integrating state-of-the-art segmentation algorithms into tracking pipelines,” *Nature Methods* 19 (2022): 829-832, https://doi.org/10.1038/s41592-022-01507-1.

42. J.-Y. Tinevez, N. Perry, J. Schindelin, G. M. Hoopes, G. D. Reynolds, E. Laplantine, S. Y. Bednarek, S. L. Shorte, K. W. Eliceiri, “TrackMate: An open and extensible platform for single-particle tracking,” *Methods* 115 (2017): 80-90, https://doi.org/10.1016/j.ymeth.2016.09.016.

43. J. Janczura, A. Weron, “Ergodicity testing for anomalous diffusion: small sample statistics,” *The Journal of Chemical Physics* 142 (2015): 144103, https://doi.org/10.1063/1.4916912.

44. C. Manzo, M. F. Garcia-Parajo, “A review of progress in single particle tracking: from methods to biophysical insights,” *Reports on Progress in Physics* 78 (2015): 124601, https://doi.org/10.1088/0034-4885/78/12/124601.
